# Efficacy of a Mobile Phone–Based Intervention on Health Behaviors and HIV/AIDS Treatment Management: Randomized Controlled Trial

**DOI:** 10.2196/43432

**Published:** 2023-04-27

**Authors:** Bach Xuan Tran, Thu Minh Bui, Anh Linh Do, Laurent Boyer, Pascal Auquier, Long Hoang Nguyen, Anh Hai Tran Nguyen, Toan Van Ngo, Carl A Latkin, Melvyn W B Zhang, Cyrus S H Ho, Roger C M Ho

**Affiliations:** 1 Institute of Preventive Medicine and Public Health Hanoi Medical University Hanoi Vietnam; 2 Bloomberg School of Public Health Johns Hopkins University Baltimore, MD United States; 3 Bach Mai Medical College Bach Mai Hospital Hanoi Vietnam; 4 Institute of Health Economics and Technology Hanoi Vietnam; 5 Research Centre on Health Services and Quality of Life Aix Marseille University Marseille France; 6 Department of Global Public Health Karolinska Institutet Stockholm Sweden; 7 Lee Kong Chian School of Medicine Nanyang Technological University Singapore Singapore Singapore; 8 Department of Psychological Medicine Yong Loo Lin School of Medicine National University of Singapore Singapore Singapore; 9 Institute for Health Innovation and Technology National University of Singapore Singapore Singapore

**Keywords:** mobile health, HIV/AIDS, treatment adherence, self-efficacy, behavior, HIV, AIDS, treatment, management, care, feasibility, efficacy, intervention, mHealth, behavior, Vietnam, application

## Abstract

**Background:**

Antiretroviral therapy (ART) is considered the most important intervention for HIV-positive patients; thus, encouraging the use of and adherence to ART are vital to HIV treatment outcomes. Advances in web and mobile technologies hold potential in supporting HIV treatment management.

**Objective:**

The aim of this study was to evaluate the feasibility and efficacy of a theory-based mobile health (mHealth) intervention on health behaviors and HIV treatment adherence among patients with HIV/AIDS in Vietnam.

**Methods:**

We performed a randomized controlled trial on 425 HIV patients in two of the largest HIV clinics in Hanoi, Vietnam. Both the intervention group (238 patients) and the control group (187 patients) received regular consultations with doctors and then participated in 1-month and 3-month follow-up visits. Patients in the intervention group received a theory-driven smartphone app to facilitate medication adherence and self-efficacy in HIV patients. Measurements were developed based on the Health Belief Model, which included the visual analog scale of ART Adherence, HIV Treatment Adherence Self-Efficacy Scale, and HIV Symptom Management Self-Efficacy Scale. We also included the 9-item Patient Health Questionnaire (PHQ-9) to assess patients’ mental health throughout treatment.

**Results:**

In the intervention group, the adherence score increased significantly (β=1.07, 95% CI .24-1.90) after 1 month, whereas the HIV adherence self-efficacy was significantly higher after 3 months (β=2.17, 95% CI 2.07-2.27) compared to the control group. There was a positive but low level of change in risk behaviors such as drinking, smoking, and drug use. Factors related to positive change in adherence were being employed and having stable mental well-being (lower PHQ-9 scores). Factors associated with self-efficacy in treatment adherence and symptom management were gender, occupation, younger age, and having no other underlying conditions. A longer duration of ART increased treatment adherence but decreased self-efficacy in symptom management.

**Conclusions:**

Our study demonstrated that the mHealth app could improve the overall ART adherence self-efficacy of patients. Further studies with larger sample sizes and longer follow-up periods are needed to support our findings.

**Trial Registration:**

Thai Clinical Trials Registry TCTR20220928003; https://www.thaiclinicaltrials.org/show/TCTR20220928003

## Introduction

For decades, HIV/AIDS has been regarded as one of the most serious public health crises in history. To date, HIV/AIDS has affected a total of 37.7 million individuals and caused 36.3 million deaths worldwide [1]. While a clinical cure for the disease is yet to be found, there have been radical advances in terms of prevention, earlier detection, diagnosis, and treatment [1]. Antiretroviral therapy (ART) is considered the most common HIV treatment, as it does not only help in suppressing viral replication but can also prevent opportunistic infections. ART has significant clinical importance for HIV treatment outcomes. For instance, people living with HIV who start ART at the age of 20 years are estimated to live 37 years longer than those not exposed to ART [2]. A study on HIV-positive children also recorded a mortality rate of 1.0 deaths per 100 child years at 4 months after ART initiation compared to 46 deaths per 100 child years in the first 4 months of other treatments [3]. As HIV/AIDS is hardly curable, ART is often a lifelong process, making adherence to treatment critical to its outcomes [4,5]. Self-efficacy in ART is defined as one’s ability to perform treatment measures such as taking medications and is considered an essential determinant of the outcome for people living with HIV/AIDS [6].

Recent advances in eHealth and mobile health (mHealth) technologies have transformed health care approaches, especially in the delivery of HIV/AIDS treatments. Lindayani et al [7] investigated the usability of a mobile-based app that provides information about HIV presentation, and Saberi et al [8] assessed the effects of another mobile app in promoting adherence to ART, as well as highlighting the feasibility of their codesigned app. Previous studies also reported 57.9% adherence to medication tracking and a 91% favorable rating of the app. Han et al [9] proposed a framework to describe the conceptualization of an HIV mobile app that includes management strategies for symptoms, health indicators, and a channel for communicating with health staff [10]. Risk indicators such as sexual activity, drug use, and alcohol use were suggested to decrease among users of HIV/AIDS mobile apps [11]. Besides highlighting the potential application of technologies in various HIV interventions, recent studies have also aimed at incorporating behavioral theories to evaluate the effectiveness of interventions [12]. A systematic review by Cho et al [13] identified common behavioral change theories applied in mobile apps in low- and middle-income countries. Behavioral change theories should be incorporated in the design of mHealth interventions with considerations of accessibility and cost-effectiveness [13]. It is important to understand the core mechanism underlying an intervention for effective transition to technological platforms [13].

Our study was developed on the Health Belief Model [14], where perceived severity and self-efficacy were measured throughout the study period and follow-up meetings for comparison, and personalized messages were sent as cues to actions. A theory-driven design helped us understand the linkages between patients’ behaviors and to identify cluster behaviors such as drinking and not working. Although mental health issues are often amplified among HIV-positive patients, not as many interventions have focused on their psychological states in app design. Therefore, our study also measured patients’ mental health symptoms as an indicator of treatment outcomes.

Although several eHealth interventions have been implemented to improve HIV treatment adherence and outcomes, there is a lack of studies performed in the settings of HIV epidemics in transition. For example, countries with large drug use populations, rapid changes in modes of transmission, and increasing coverage of smartphone use require specific and contextualized evidence and policies. In Vietnam, it is estimated that 250,000 individuals, including both adults and children, are living with HIV, 169,000 of whom are receiving ART [15]. Although mHealth apps are effective tools to complement existing care, to date, no study in Vietnam has attempted to investigate the potential of mobile-based interventions for individuals living with HIV [16]. Therefore, the aim of this study was to evaluate the feasibility and efficacy of a theory-based mHealth intervention on HIV treatment adherence, self-efficacy, and health behaviors among people living with HIV in Vietnam.

## Methods

### Study Design and Participants

This was a randomized controlled trial including participants recruited from outpatient HIV clinics at Bach Mai Hospital and Ha Dong General Hospital from March 2018 to December 2019. These are among the most popular hospitals for HIV treatment in Hanoi, Vietnam, and are located in different parts of Hanoi to ensure geographical variety and different levels of administration. The Bach Mai HIV Outpatient Clinic (a central-special hospital) is funded by the government to provide ART to nearly 3000 HIV-positive patients in Hanoi, while the HIV Clinic in Ha Dong General Hospital (provincial-level hospital) provides insurance-covered testing and treatment as well as free counseling. Patients were excluded if they were using any other HIV-assist smartphone app at the time of the study, or had cognitive impairments or disabilities that would hinder the use of our app. Convenience sampling was used in the selection of participants. All participants agreed to participate in this study by signing a written informed consent form. By the end of the sample recruitment period, a total of 495 patients were recruited and a baseline assessment was performed [17,18]. Participants were then randomized into the intervention arm (n=248) and the control arm (n=247) by a computer software. After the randomization phase, 65 participants were excluded, including 5 from the intervention arm and 60 from the control arm with the following reasons: family issues (n=2), transferred to other clinics (n=3), used other HIV-assist smartphone apps (n=55), stopped participating in the study (n=2), and technical problems with the smartphone (n=4). A total of 430 HIV patients fulfilled the inclusion criteria and participated in the intervention phases, including 243 patients in the intervention arm and 187 patients in the control arm. Five patients in the intervention group did not complete regular follow-ups after 1 month of the intervention and were excluded. Finally, data of 238 patients in the intervention arm and 187 patients in the control arm were analyzed.

**Figure 1:**
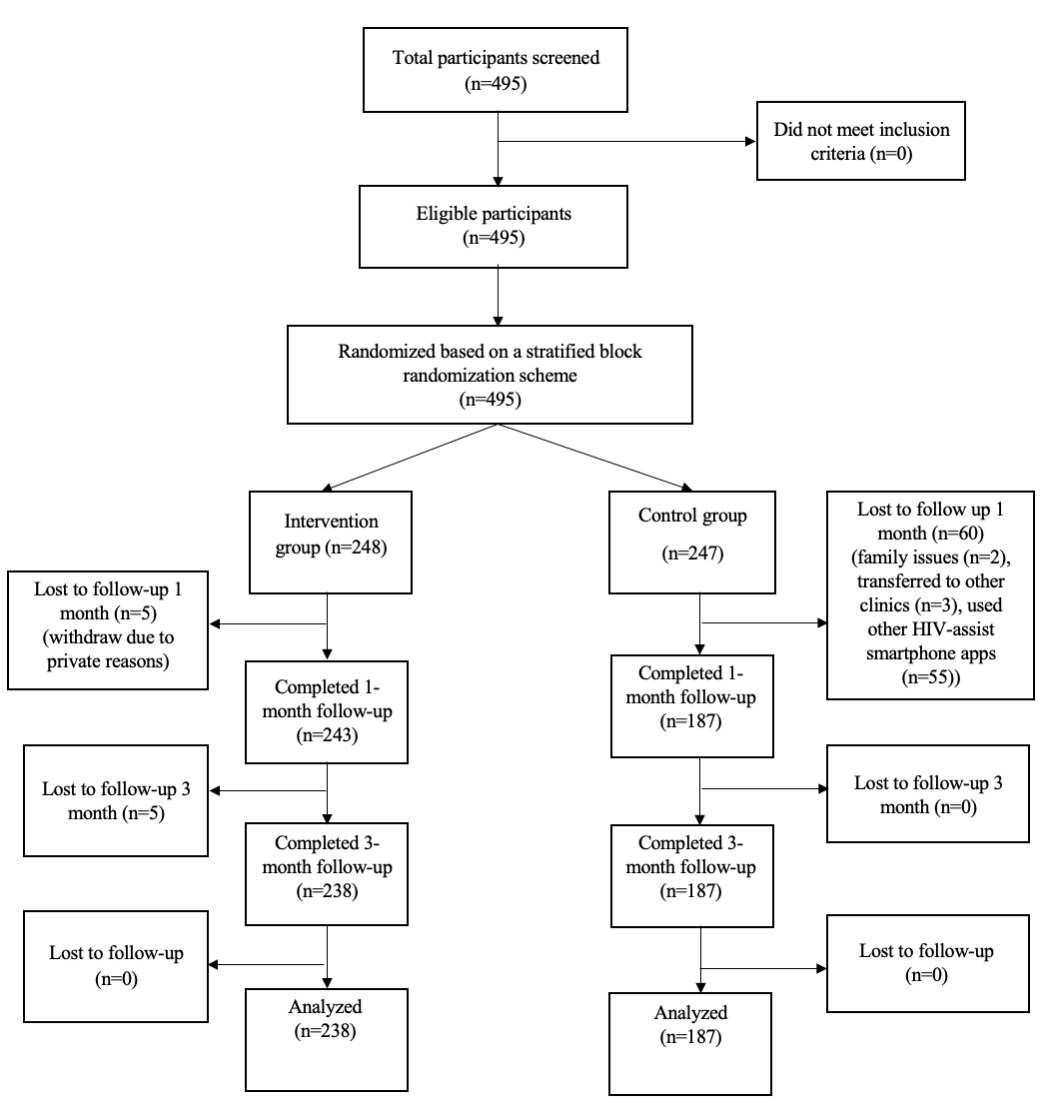
Study flowchart.

Figure 1 illustrates the flow chart of this trial. No difference was found between included and excluded patients regarding gender (*P*=.45), occupation (*P*=.08), hazardous drinking (*P*=.36), and medication adherence (*P*=.11) at baseline. However, significant differences were found in age (*P*<.001) education (*P*=.003), marital status (*P*=.009), smoking (*P*=.02), duration of ART (*P*<.001), and CD4 initiation (*P*=.03).

### Experimental Procedure

Participants in both the intervention and control groups received regular consultations from doctors at the clinic per Vietnam’s guidelines for HIV/AIDS patient care. Patients in the intervention group received a theory-based smartphone app (eCARE app) for promoting medication adherence and self-management in HIV patients. Participants undertook the assigned intervention immediately after performing the baseline survey.

### App Design

#### Overview

The eCARE app was developed based on the Analysis, Design, Development, Implementation, and Evaluation (ADDIE) model. We first performed a literature review to build the content components for the app. The messages of the app were developed based on Social Cognitive Theory, the Health Belief Model, and the Integrated Theory of Behavior Change, which were adapted into personalized messages sent to the patients by recognizing their current states of behavioral changes. The app included a GIS-based map of HIV medical facilities, as well as other mental health and harm reduction services. The app was developed by arranging the necessary information, editing the text, and adding appropriate illustrations. The first version was designed as a web-based simulator and evaluated by experts in the field of HIV/AIDS treatment as well as by some patients in the study population. After fixing the bugs, the final version (in terms of content) was developed and put into beta form. The testing phase included stability testing on iOS and Android platforms and testing the effectiveness of the app. The trial phase was conducted on 20 patients to investigate their requirements for the app in terms of form and content. The intervention group was given access to the eCARE app through medical staff at the treatment facility.

**Figure 2:**
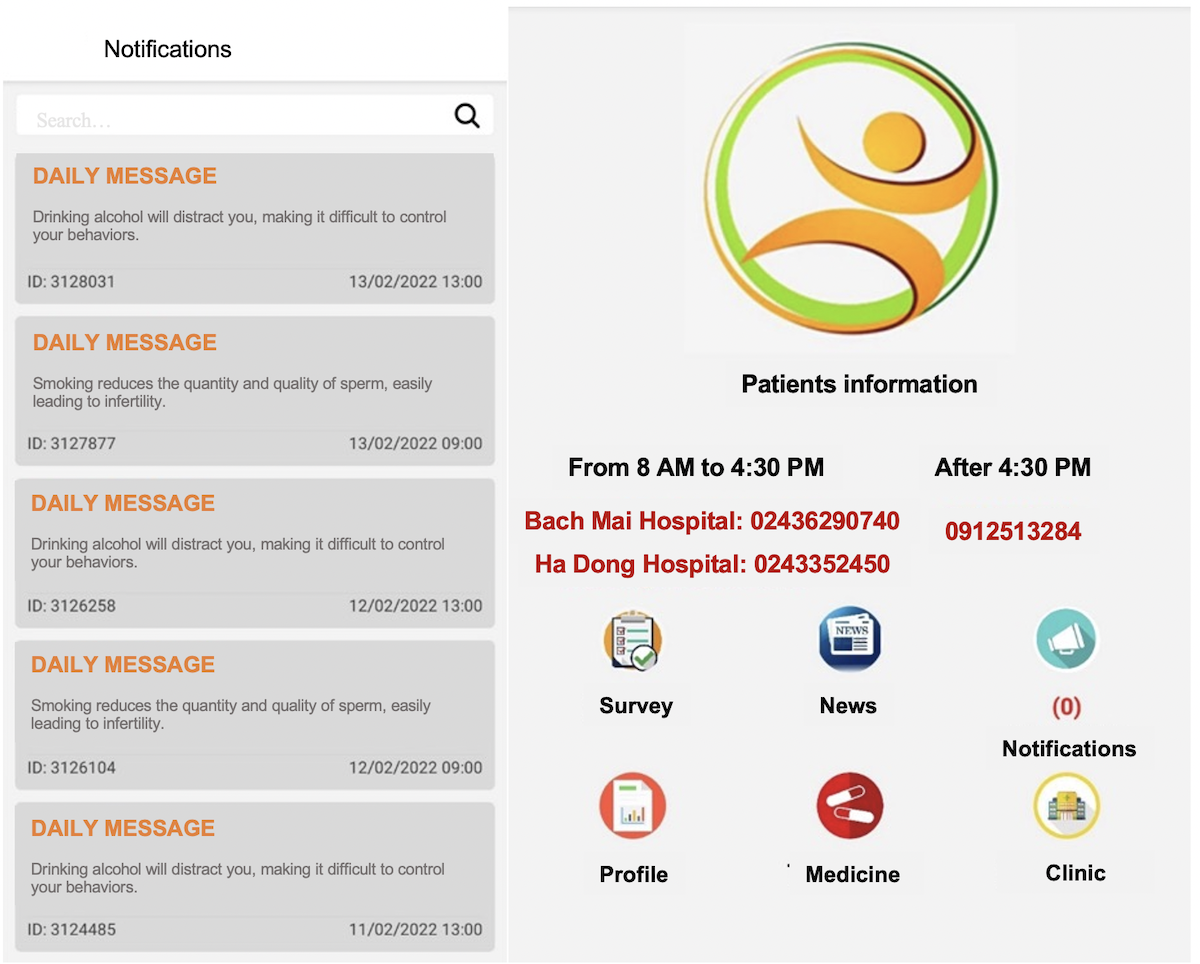
Main functions of the eCARE smartphone app (in English).

The final smartphone app (eCARE) contains the following core functions: (1) personal medical record; (2) medication reminder; (3) behavior monitoring (including tobacco smoking, alcohol drinking, and illegal substance use); (4) connection to health facilities; (5) guidelines, information, and news; and (6) contact to HIV clinics (Table 1). Figure 2 illustrates the main functions of the app.

**Table 1:** Components and theories informing the design of the eCARE app.

Theory	Application	Design of functions in the mobile app	Elements of the intervention
Social Cognitive Theory	Social acquisition of knowledge results in behavior changes	Behavior monitoring; guidelines, information, and news	The app evaluates the knowledge of patients on HIV treatment and selects relevant messages to improve their knowledge and inform behavior changes
Health Belief Model	People behave in a way to minimize what they perceive as threats and to maximize what are perceived as benefits	Behavior monitoring; connection to health facilities; guidelines, information, and news	Regularly evaluate and improve patients’ perceived susceptibility, perceived seriousness, perceived benefits, and perceived barriers by sending contextualized messages
Integrated Theory of Behavior Change	Improving knowledge, beliefs, self-regulation skills and abilities, and social facilitation	Medication reminder; behavior monitoring; connection to health facilities; guidelines, information, and news; and contact to HIV clinics	Generate supporting messages in different time frames, generating reminders to take pills, linking patients with clinics and counselors

#### Personal Medical Record

This section retrieves and consolidates all the information from the respective medical databases to create a centralized medical record for the patient. This approach can avoid missing information that might hinder future health care interventions. Patients are provided with an overview of their medical information and their treatment plans.

#### Medication Reminder

The medication reminder provides simple daily reminders to take medication, which ultimately improves adherence to ART. The reminders are set as alarms or sent as text messages at designated times. This function also allows health care staff to track and monitor patients’ compliance.

#### Behavior Monitoring (Tobacco Smoking, Alcohol Drinking, and Illegal Substance Use)

The behavior monitoring functionality allows individuals to self-report their behaviors daily. The app disseminates a variety of messages (through phone messages, app notifications, and videos) to help individuals change their unhealthy behaviors. These messages are developed based on the Social Cognitive Theory and the Health Belief Model. Each message is also individualized according to the baseline information of the patient such as gender, age, location, smoking, alcohol drinking, substance use, health status, and medication adherence (eg, “Let yourself and your loved ones be proud that you have quit smoking for a healthy life” or “Adhering to medication is the only way to prevent HIV from progressing”).

#### Guidelines, Information, and News

News and guidelines about HIV/AIDS treatment and relevant issues are provided within the app and are updated on a daily basis.

#### Contact to HIV Clinics

This function allows users to interact directly with medical staff when they need health advice. In emergencies, individuals will be connected to the nearest medical facility.

### Data Collection and Measurement

The following data were obtained from participants: (1) demographic and patient characteristics, (2) risk behaviors, (3) visual analog scale (VAS) of ART Adherence, (4) HIV Treatment Adherence Self-Efficacy Scale (HIV-ASES), and (5) HIV Symptom Management Self-Efficacy Scale. Before collecting data, the questionnaire was piloted in a group of HIV patients to resolve logical issues and adjust questions to improve accuracy. A face-to-face interview of 15-20 minutes was conducted in a closed room to ensure privacy and limit outside influences. Trained staff members assisted in data collection. These data were collected at baseline and at 1-month and 3-month follow-ups during their ART medication visits with doctors. 

Participants were asked about three risk behaviors, including drinking, smoking, and drug use, at baseline. At the 3-month follow-up, they were further asked “Compared to before participating in the study, how do you assess your drinking/smoking/drug use now?” with three answer options (increase, constant, decrease) to explore the change of the participant’s risk behaviors.

The VAS of ART Adherence is a self-reported scale that was used to assess adherence to ART treatment among participants; scores range from 0 (nonadherent) to 100 (completely adherent) [19]. Patients who had higher scores indicated higher adherence to treatment plans.

The HIV-ASES includes 12 items and is used to assess patients’ confidence in adherence to treatment plans, including medications, diet, exercise, and the consumption of vitamins. Scores range from 0 (cannot do at all) to 10 (certainly able to do) [20,21]. The Cronbach α was .98 indicating good reliability.

The HIV Symptom Management Self-Efficacy Scale has a total of 10 items adapted from the abbreviated 6-item Chronic Disease Self-Efficacy Scale. The scale evaluates four domains, including symptom control, role function, emotional function, and communication with physicians. This scale measures the confidence in patients’ capacity to manage HIV symptoms. Scores range from 1 (not at all confident) to 10 (completely confident). The total score is calculated as the sum of means; higher scores indicate higher confidence levels [22]. The Cronbach α was .99.

Although these two scales, the HIV-ASES and the HIV Symptom Management Self-Efficacy Scale, are important measures to assess patients’ readiness and capacity to initiate treatment, after the pilot trial, we found that these scales were not able to accurately reflect the change in the first month of ART among patients at Bach Mai Hospital, resulting in a very high ceiling effect with almost all patients showing the highest scores. Thus, we decided not to apply these scales in the 1-month follow-up. The reason was that Bach Mai Hospital is the largest special-level hospital in Vietnam; thus, the procedure for initiating treatment was implemented very comprehensively. The patients’ readiness was improved substantially by various interventions, including family, group, and individual counseling; peer support; health checkups; and opportunistic infection treatment.

Demographic and HIV patient characteristics were included as covariates for analysis. We asked participants to report their background information, including gender, age, education, occupation, and marital status. Some characteristics related to health status and HIV patient characteristics were also collected such as the CD4 cell count at treatment initiation (cells/µL), duration of ART (years), and the presence of comorbidities/acute symptoms.

Patients’ mental health status was assessed with the 9-item Patient Health Questionnaire (PHQ-9), including 9 questions that assess depressive symptoms over the last 14 days. For each question, participants were asked to respond on a 4-point Likert scale ranging from 0 to 3 (0, not at all; 1, several days; 2, more than half the days; and 3, nearly every day). The total score ranges from 0 to 27, with scores above 10 being categorized as depression [23,24]. The Cronbach α of the questionnaire was previously reported to be .86 [25]. In this study, the Cronbach α of the PHQ-9 was excellent at .94.

In addition, the overall study included quality of life assessments as a measure of the impact of this intervention; however, this outcome is presented in a separate analysis. In this manuscript, we focus on immediate outputs to examine the efficacy of the intervention.

### Statistical Analysis

STATA version 16.0 was used for the descriptive and analytical statistical analyses. To handle missing data, we used the listwise deletion method to clean the data before analysis. For comparison of the change in patients’ health behaviors and subgroups, we used the Wilcoxon rank-sum test; the Kruskal-Wallis test was used for comparisons of continuous variables and the *χ*^2^ test was used for comparisons of nominal variables. To determine the effect of the intervention, we tested the primary hypothesis that participants in the intervention group would have higher VAS of ART Adherence, HIV-ASES, and HIV Symptom Management Self-Efficacy Scale scores when compared to those of patients in the control group from baseline to follow-up at 1 and 3 months.

Potential covariates for full models of change in risk behaviors among participants included individual characteristics, health status, and adherence. To test our hypotheses, we used multilevel mixed effects linear regression analysis to account for correlations of repeated-measures data for all these scales. Multivariate logistic regression was used to determine factors associated with the change level of the three risk behaviors (drinking, smoking, and drug use). A *P* value <.05 was considered statistically significant.

### Ethical Considerations

The protocol of this study was approved by the institutional review board of Hanoi Medical University (code: 18NCS17/HDDDDHYHN). All participants agreed to participate in this study through a written informed consent form. Those invited to participate in the study were fully explained the content, purpose, and benefits of participating. There was no risk to the patients participating in this study. The information they provided was completely anonymous. The researchers and staff participating in the study signed a commitment not to disclose information collected during the study without the consent of the participants. The collected information is kept confidential and is only used for research purposes, not for other purposes. The data are encrypted to ensure confidentiality of the information.

## Results

The main participant characteristics are summarized in Table 2; the full data table is presented in Multimedia Appendix 1. The mean age was 38.6 years and 40.2% of the participants were women. In particular, compared with the control group, more participants in the intervention group indicated having a high school or below education (69.4% vs 55.1%) and being married (73.2% vs 60.9%). Additionally, the duration of ART (7.8 years vs 6.1 years) and age (40.9 years vs 36.9 years) of the control group were higher than those of the intervention group. There were significant differences (all *P*<.05) between people belonging to the intervention group and control group regarding education, marital status, age, and duration of ART. No differences at baseline were found between the two groups regarding gender, occupation, CD4 cell count, hazardous drinking, drug use/injection, and ART adherence (all *P*>.05).

The change of three aspects, including adherence (VAS), HIV treatment adherence self-efficacy, and symptom management self-efficacy, are presented in Table 3. Overall, a significantly higher score of all aspects was observed from baseline to the 3-month follow-up. At baseline, the control group had a higher score in all three aspects. In terms of adherence (VAS), the score of the intervention group was slightly lower than that of the control group (75.2 vs 75.8) at baseline. When compared to the baseline stage, the change of adherence score in the intervention group (increasing from 18.5 points at the 1-month follow-up to 19.7 points at the 3-month follow-up) was higher than that of the control group (increasing from 16.8 points at the 1-month follow-up to 19.2 points at the 3-month follow-up). Similarly, the scores of HIV treatment adherence self-efficacy and HIV symptom management self-efficacy were 95.8 and 71.9, respectively, at the baseline stage, which were lower than those of the control group. At the 3-month follow-up, compared to the score at baseline, the intervention group showed a greater increase than the control group. These differences between baseline and 1-month or 3-month follow-up were significantly different (Table 3).

Table 4 describes the change in risk behaviors at the 3-month follow-up among participants. Generally, a higher proportion of patients in the intervention group showed positive changes in drinking and smoking behavior compared with the control group at the 3-month follow-up. In the intervention group, 41.2% of participants reporting drinking behavior and 15.2% reporting smoking behavior, indicating that that the level of these behaviors had decreased, compared to 33.7% and 10.2% in the control group, respectively. Regarding drug use behavior, at the 3-month follow-up, 28.1% of participants reported drug use behavior, indicating that this behavior had decreased.

Table 5 shows that treatment adherence according to the VAS tended to increase after 1 and 3 months in both the intervention and control groups. In the intervention group, the VAS score increased significantly after 1 month compared with that of the control group. Similarly, after 3 months, the total scores of self-assessment of adherence effectiveness and effectiveness of HIV symptom management both tended to increase. However, the HIV Symptom Management Self-Efficacy score in the intervention group after 3 months was significantly lower than that of the control group.

Table 6 presents factors related to the change in risk behaviors among participants. Women had lower odds of decreasing their level of drinking, smoking, and drug use behaviors than men. Moreover, people having a stable job were less likely to decrease their level of drug use. By contrast, people with a higher PHQ-9 score tended to have higher odds of decreasing their level of drug use. Older participants were more likely to exhibit a greater decrease in their level of drinking. A higher score for HIV treatment adherence self-efficacy was associated with a higher odds of decreasing the level of drinking as well as smoking.

**Table 2:** Socioeconomic characteristics and health status of participants.

Characteristics	Intervention group (n=238), mean (SD)	Control group (n=187), mean (SD)	Total (N=425), mean (SD)	*P* value
Age (years)	36.9 (7.4)	40.9 (7.0)	38.6 (7.5)	<.001
Duration of ART^a^ (years)	6.1 (3.4)	7.8 (3.1)	6.8 (3.3)	<.001
CD4 count at treatment initiation (cells/µL)	230.7 (204.7)	206.4 (199.6)	220.4 (202.8)	.03
PHQ-9^b^ score (range 0-27)	3.9 (3.5)	4.1 (3.3)	3.9 (4.4)	.88

^a^ART: antiretroviral therapy.

^b^PHQ-9: 9-item Patient Health Questionnaire.

**Table 3:** Change of adherence, HIV treatment adherence self-efficacy, and symptom management self-efficacy among HIV patients at 1 and 3 months after starting the intervention.

Measures		Baseline, mean (SD)	1-month follow-up				3-month follow-up		
			Mean (SD)	Difference from baseline	*P* value	Mean (SD)		Difference from baseline	*P* value
**Adherence (VAS^a^) (range 0-100)**									
	Intervention group	75.2 (10.7)	93.7 (7.7)	18.5	<.001	94.9 (7.4)		19.7	<.001
	Control group	75.8 (16.8)	92.6 (11.1)	16.8	<.001	95.0 (8.4)		19.2	<.001
	*P* value	.11	.91	—^b^	—	.19		—	—
**HIV Treatment Adherence Self-Efficacy Scale (range 0-120)**									
	Intervention group	95.8 (22.4)	—	—	—	100.5 (21.8)		4.7	.009
	Control group	99.6 (22.9)	—	—	—	103.0 (20.1)		3.4	.11
	*P* value	.10	—	—	—	.28		—	—
**HIV Symptom Management Self-Efficacy Scale (range 0-100)**									
	Intervention group	71.9 (22.7)	—	—	—	77.1 (20.3)		5.2	.001
	Control group	72.5 (21.7)	—	—	—	76.8 (19.5)		4.3	.009
	*P* value	.89	—	—	—	.46		—	—

^a^VAS: visual analog scale.

^b^Not applicable.

**Table 4:** Level of change of risk behaviors at the 3-month follow-up.

Characteristics		Intervention group (n=238), n (%)	Control group (n=187), n (%)	Total (N=425), n (%)	*P* value	
**Drinking**						.24
	Constant/increase	77 (58.8)	67 (66.3)	144 (62.1)		
	Decrease	54 (41.2)	34 (33.7)	88 (37.9)		
**Smoking**						.22
	Constant/increase	134 (84.8)	106 (89.8)	240 (87.0)		
	Decrease	24 (15.2)	12 (10.2)	36 (13.0)		
**Drug use**						.93
	Constant/increase	65 (72.2)	58 (71.6)	123 (71.9)		
	Decrease	25 (27.8)	23 (28.4)	48 (28.1)		

**Table 5:** Multilevel mixed effects linear regression to identify factors related to change in adherence and symptom management self-efficacy among participants.

Characteristics				Adherence (VAS^a^)					HIV Treatment Adherence Self-Efficacy					HIV Symptom Management Self-Efficacy			
				β (95% CI)		*P* value		β (95% CI)			*P* value		β (95% CI)			*P* value	
**Individual characteristics**																	
	Group (intervention vs control=reference)			–1.16 (–1.69 to –.63)		<.001		–6.91 (–7.22 to –6.61)			<.001		.28 (.16 to .40)			<.001	
	**Time of study (baseline=reference)**																
		1-month follow-up	11.80 (10.47 to 13.12)		<.001		—^b^			—^b^		—^b^			—^b^		
		3-month follow-up	12.08 (9.33 to 14.83)		<.001		–8.95 (–15.28 to –2.62)			.006		2.80 (2.29 to 3.31)			<.001		
	**Time×group (control group×time=reference)**																
		Intervention group after 1 month	1.07 (.24 to 1.90)		.01		—^b^			—^b^		—^b^			—^b^		
		Intervention group after 3 months	.19 (–.05 to .43)		.11		2.17 (2.07 to 2.27)			<.001		–.43 (–.77 to –.08)			.02		
	Gender (female vs male=reference)			–.16 (–1.28 to .95)		.77		–2.92 (–5.22 to –.62)			.01		3.13 (2.24 to 4.02)			<.001	
	Marital status (married vs other= reference)			–.55 (–1.69 to .59)		.34		–.82 (–2.55 to .90)			0.35		2.39 (.89 to 3.90)			.002	
	Occupation (stable job vs unstable job= reference)			.62 (.51 to .74)		<.001		5.82 (5.47 to 6.18)			<.001		1.06 (1.04 to 1.07)			<.001	
	Age (unit: year)			–.03 (–.14 to .08)		.60		–.29 (–.36 to –.22)			<.001		.005 (–.11 to .12)			.94	
**Health status**																	
	Comorbidities/acute symptoms (yes vs no=reference)			–2.65 (–7.20 to 1.91)		.25		–8.53 (–13.54 to –3.52)			.001		–2.11 (–3.80 to –.41)			.02	
	Duration of ART^c^ (unit: year)			.01 (–.22 to .24)		.94		.23 (.04 to .42)			.02		–.69 (–1.03 to –.35)			<.001	
	Mental well-being (PHQ-9^d^) (unit: score)			–.63 (–.86 to –.41)		<.001		–1.14 (–1.72 to –.55)			<.001		–.17 (–.25 to –.09)			<.001	
	ART initiation (cell/µL) (<200=reference)			.001 (–.003 to .004)		.76		–.01 (–.01 to –.001)			.02		–.01 (–.01 to –.004)			<.001	

^a^VAS: visual analog scale.

^b^Not applicable.

^d^PHQ-9: 9-item Patient Health Questionnaire.

**Table 6:** Logistic regression to identify factors related to change in risk behaviors among participants.

Characteristics				Drinking (decrease vs constant/increase)				Smoking (decrease vs constant/increase)					Drug use (decrease vs constant/increase)		
				OR^a^ (95% CI)		*P* value		OR (95% CI)		*P* value		OR (95% CI)			*P* value
**Individual characteristics**															
	Group (intervention vs control=reference)			1.96 (0.91-4.23)		.08		1.60 (0.53-4.86)		.41		1.31 (0.42-4.08)			.64
	Education (college/tertiary and above vs high school and below=reference)			0.95 (0.45-2.02)		.90		1.00 (0.38-2.62)		.99		0.24 (0.05-1.04)			.06
	Gender (women vs men=reference)			0.06 (0.02-0.21)		<.001		0.04 (0.00-0.35)		.004		0.04 (0.00-0.34)			.003
	Marital status (married vs other=reference)			0.53 (0.20-1.37)		.19		0.49 (0.15-1.57)		.23		3.79 (0.80-17.88)			.09
	Occupation (stable job vs unstable job=reference)			1.24 (0.61-2.53)		.55		1.43 (0.54-3.78)		.48		0.15 (0.04-0.56)			.005
	Age (unit: year)			1.10 (1.03-1.17)		.004		1.05 (0.96-1.13)		.27		0.96 (0.88-1.06)			.44
**Health status**															
	Comorbidities/acute symptoms (yes vs no=reference)			0.80 (0.38-1.69)		.55		1.02 (0.37-2.81)		.98		0.35 (0.11-1.14)			.08
	Duration of ART^b^ (unit: year)			0.97 (0.86-1.10)		.65		1.07 (0.91-1.25)		.40		0.97 (0.80-1.17)			.72
	Mental well-being (PHQ-9^c^) (unit: score)			1.06 (0.99-1.13)		.10		0.94 (0.85-1.04)		.21		1.19 (1.07-1.32)			.001
	**CD4 count at ART initiation (cells/µL) (<200=reference)**														
		201-350	1.49 (0.66-3.37)		.34		1.77 (0.61-5.15)		.30		2.89 (0.83-10.04)			.10	
		351-500	1.07 (0.40-2.83)		.90		0.32 (0.06-1.85)		.20		3.42 (0.78-15.08)			.10	
		>500	2.43 (0.64-9.20)		.19		0.83 (0.13-5.39)		.85		1.26 (0.11-14.10)			.85	
**Adherence**															
	HIV Symptom Management Self-Efficacy Scale (unit: score)			0.99 (0.97-1.00)		.09		0.97 (0.95-0.99)		.002		1.00 (0.98-1.03)			.76
	HIV Treatment Adherence Self-Efficacy Scale (unit: score)			1.02 (1.00-1.03)		.02		1.02 (1.00-1.04)		.04		1.00 (0.98-1.02)			.73

^a^OR: odds ratio.

^b^ART: antiretroviral therapy.

^c^PHQ-9: 9-item Patient Health Questionnaire.

## Discussion

### Principal Findings

This study assessed the effectiveness of an evidence-based codesigned mobile intervention for individuals living with HIV. Our study revealed that those who used the app had increased adherence and a higher level of self-efficacy in terms of symptom management. However, the effectiveness of this app in changing risk behaviors such as smoking, alcohol use, and drug use was limited.

Our study supports the main hypothesis that individuals who used the intervention have better adherence at follow-up appointments. This finding is congruent with other studies about mHealth in HIV [8,9], and in other diseases such as breast cancer [26] and hypertension [27]. However, it is important to identify factors that directly contribute to treatment adherence, as this information would be paramount for future app design and the conceptualization of mHealth for other disorders. A systematic review by Pérez-Jover et al [28] identified factors that lead to increased adherence, including an easy-to-navigate design, useful information, and relevant features. One crucial similarity shared by effective apps was that they utilized a codesign approach during the conceptualization phase. The utilization of these methods ensured that the final product was relevant and could cater to the specific needs of patients. Some additional features that were found to increase adherence to medications/treatment included reminders with alarms, information about medications, and medication-tracking functions.

In our study, the change in adherence was significantly higher in the intervention group than in the control group at 1 month into the intervention but not at 3 months. This could be explained by the fact that adherence is required in HIV/AIDS treatment; thus, it is mandatory for patients to improve compliance regardless of their intervention status. Moreover, the high educational level of the control group could have resulted in higher compliance even without the use of our smartphone app. Although the difference in adherence between the two groups was not significant, the level of treatment adherence self-efficacy was significantly higher in the intervention group than in the control group at 3 months after starting the intervention. In existing literature, self-efficacy was significantly correlated with short- and long-term adherence [21,29,30]. Thus, this smartphone app also enhanced the sustainability of HIV/AIDS treatment. Longitudinal studies with longer follow-up periods are needed to test this hypothesis. In addition, it should be noted that the level of HIV symptom management self-efficacy was reduced at 3 months in the intervention group compared with that of the control group. This phenomenon may reflect a downside of our app, as patients may become dependent on the app to manage their health status and treatment. A further qualitative study is needed to evaluate and propose solutions to this issue.

This study managed to test out a new mobile intervention for individuals living with HIV that was conceptualized from a theoretical approach. The data from our study justified the feasibility and acceptability of this new app and complemented existing data regarding mobile interventions for people living with HIV. For example, Garg et al [31] examined a peer-customized mobile app that was based on the principle of self-learning and reported that the app improved HIV-related knowledge from 20% to 60%. Sullivan and Hightow-Weidman [32] reported that core functions of mobile apps could serve as an interventional tool individually and that the apps could not only provide rich media coverage but also serve as a platform to tailor interventions for personal use.

### Strengths and Limitations

The main strengths are that this study represents the first effort of evaluating an mHealth app in the field of HIV/AIDS in Vietnam. This study managed to test a new mobile intervention for individuals living with HIV that was conceptualized from a theoretical approach. We utilized codesign methods and theoretical models in the conceptualization of the assessed mobile app.

Certain limitations also exist in our study. With respect to the confidentiality of HIV diagnosis and medical records, we were not able to preidentify the survey sample. Instead, the sample of participants was identified using convenience sampling and was subsequently randomized. The randomization method could not remove all underlying differences among participants assigned to the experimental or control conditions. There were also differences in educational levels, marital status, age, and duration of ART between the two groups. In addition, we acknowledge that ART outcomes will reduce transmission as well. However, we did not include sexual behaviors in our assessments, as we realized during the pilot trial that this remains a sensitive topic among Vietnamese people living with HIV, which could have affected the compliance to the app-based intervention since they were afraid of being traced and a loss of confidentiality. Furthermore, the follow-up period in our study was 3 months, while the ideal follow-up period should be 6 months or longer to fully determine the sustainability of treatment improvements.

### Conclusion

Our study demonstrated the effectiveness of an mHealth app in the overall treatment adherence and self-efficacy to ART among people living with HIV in Vietnam. It is evident that individuals who used the intervention have better adherence at follow-up appointments, and therefore mHealth interventions should continue to be developed. However, further studies with larger sample sizes and longer follow-up periods are recommended to validate and extend our findings.

## Appendix

Multimedia Appendix 1 Full data on socioeconomic characteristics and health status of participants.

Multimedia Appendix 2 CONSORT-eHealth checklist (V1.6.1).

## Abbreviations

ADDIE: Analysis, Design, Development, Implementation, and Evaluation
ART: antiretroviral therapy
HIV-ASES: HIV Treatment Adherence Self-Efficacy Scale
mHealth: mobile health
PHQ-9: 9-item Patient Health Questionnaire
VAS: visual analog scale

## Data Availability

The data of this study cannot be made publicly available due to the restriction of the Vietnam HIV/AIDS Prevention and Control Law. Reasonable requests for data should be sent to the corresponding author.
