# Supplementary material for: Efficacy of a Mobile Phone–Based Intervention on Health Behaviors and HIV/AIDS Treatment Management: Randomized Controlled Trial
Source: J Med Internet Res. 2023 Apr 27;25:e43432. doi: 10.2196/43432 (PMC10176129; doi:10.2196/43432)
Supplement: Multimedia Appendix 1 [file jmir_v25i1e43432_app1.docx]

**Appendix 1: Full data on socioeconomic characteristics and health status of participants**

|  | **Intervention group** | | **Control group** | | **Total** | | ***P* value** |
| --- | --- | --- | --- | --- | --- | --- | --- |
|  | **n** | **%** | **n** | **%** | **n** | **%** |  |
| **Total** | 243 | 57.2 | 182 | 42.8 | 425 | 100.0 |  |
| **Gender** |  |  |  |  |  |  |  |
| Male | 149 | 61.3 | 105 | 57.7 | 254 | 59.8 | .45 |
| Female | 94 | 38.7 | 77 | 42.3 | 171 | 40.2 |  |
| **Education** |  |  |  |  |  |  |  |
| High school and below | 130 | 55.1 | 125 | 69.4 | 255 | 61.3 | .003 |
| College/Tertiary and above | 106 | 44.9 | 55 | 30.6 | 161 | 39.0 |  |
| **Marital status** |  |  |  |  |  |  |  |
| Married | 143 | 60.9 | 131 | 73.2 | 274 | 66.2 | .009 |
| Others | 92 | 39.2 | 48 | 26.8 | 140 | 33.8 |  |
| **Occupation** |  |  |  |  |  |  |  |
| Stable jobs | 90 | 37.2 | 82 | 45.6 | 172 | 40.8 | .08 |
| Unstable jobs | 152 | 62.8 | 98 | 54.4 | 250 | 59.2 |  |
| **Smoking** |  |  |  |  |  |  |  |
| No | 208 | 85.6 | 172 | 93.0 | 480 | 88.8 | .02 |
| Yes | 35 | 14.4 | 13 | 7.3 | 58 | 11.2 |  |
| **Hazardous drinking** |  |  |  |  |  |  |  |
| No | 218 | 89.7 | 168 | 92.3 | 386 | 90.8 | .36 |
| Yes | 25 | 10.3 | 14 | 7.7 | 39 | 9.2 |  |
|  | **Mean** | **SD** | **Mean** | **SD** | **Mean** | **SD** | **p-value** |
| **Age** | 36.9 | 7.4 | 40.9 | 7.0 | 38.6 | 7.5 | <.001 |
| **Duration of ART**^a^ (years) | 6.1 | 3.4 | 7.8 | 3.1 | 6.8 | 3.3 | <.001 |
| **CD4 initiation (cell/µL)** | 230.7 | 204.7 | 206.4 | 199.6 | 220.4 | 202.8 | .03 |
| **Adherence (VAS**^b^**) (range 0-100)** | 75.2 | 10.7 | 75.8 | 16.8 | 75.5 | 13.6 | .11 |

^a^ART: antiretroviral therapy.

^b^VAS: visual analog scale.
