# Supplementary material for: Efficacy of a Mobile Phone–Based Intervention on Health Behaviors and HIV/AIDS Treatment Management: Randomized Controlled Trial
Source: J Med Internet Res. 2023 Apr 27;25:e43432. doi: 10.2196/43432 (PMC10176129; doi:10.2196/43432)
Supplement: Multimedia Appendix 2 [file jmir_v25i1e43432_app2.pdf]

CONSORT-EHEALTH (V 1.6.1) - Submission/Publication Form

The CONSORT-EHEALTH checklist is intended for authors of randomized trials evaluating web-based and Internet-based applications/interventions, including mobile interventions, electronic games (incl multiplayer games), social media, certain telehealth applications, and other interactive and/or networked electronic applications. Some of the items (e.g. all subitems under item 5 - description of the intervention) may also be applicable for other study designs.

The goal of the CONSORT EHEALTH checklist and guideline is to be
a) a guide for reporting for authors of RCTs,
b) to form a basis for appraisal of an ehealth trial (in terms of validity)

CONSORT-EHEALTH items/subitems are MANDATORY reporting items for studies published in the Journal of Medical Internet Research and other journals / scientific societies endorsing the checklist.

Items numbered 1., 2., 3., 4a., 4b etc are original CONSORT or CONSORT-NPT (non-pharmacologic treatment) items.
Items with Roman numerals (i., ii, iii, iv etc.) are CONSORT-EHEALTH extensions/clarifications.

As the CONSORT-EHEALTH checklist is still considered in a formative stage, we would ask that you also RATE ON A SCALE OF 1-5 how important/useful you feel each item is FOR THE PURPOSE OF THE CHECKLIST and reporting guideline (optional).

Mandatory reporting items are marked with a red *.
In the textboxes, either copy & paste the relevant sections from your manuscript into this form - please include any quotes from your manuscript in QUOTATION MARKS,
or answer directly by providing additional information not in the manuscript, or elaborating on why the item was not relevant for this study.

YOUR ANSWERS WILL BE PUBLISHED AS A SUPPLEMENTARY FILE TO YOUR PUBLICATION IN JMIR AND ARE CONSIDERED PART OF YOUR PUBLICATION (IF ACCEPTED).
Please fill in these questions diligently. Information will not be copyedited, so please use proper spelling and grammar, use correct capitalization, and avoid abbreviations.

DO NOT FORGET TO SAVE AS PDF _AND_ CLICK THE SUBMIT BUTTON SO YOUR ANSWERS ARE IN OUR DATABASE !!!

Citation Suggestion (if you append the pdf as Appendix we suggest to cite this paper in the caption):
Eysenbach G, CONSORT-EHEALTH Group
CONSORT-EHEALTH: Improving and Standardizing Evaluation Reports of Web-based and Mobile Health Interventions
J Med Internet Res 2011;13(4):e126
URL: http://www.jmir.org/2011/4/e126/
doi: 10.2196/jmir.1923
PMID: 22209829

bach.ipmph2@gmail.com Chuyển đổi tài khoản

Không được chia sẻ

Đã lưu bản nháp

* Biểu thị câu hỏi bắt buộc

Your name *

First Last

Bach Xuan Tran

Primary Affiliation (short), City, Country *

University of Toronto, Toronto, Canada

Hanoi Medical University, Hanoi, Vietnam

Your e-mail address *

abc@gmail.com

bach.ipmph2@gmail.com

Title of your manuscript *

Provide the (draft) title of your manuscript.

Efficacy of a mobile phone-based intervention on health behaviors and HIV/AIDS treatment management: a Randomized Controlled Trial

Name of your App/Software/Intervention *

If there is a short and a long/alternate name, write the short name first and add the long name in brackets.

eCARE

Evaluated Version (if any)

e.g. "V1", "Release 2017-03-01", "Version 2.0.27913"

Câu trả lời của bạn

Language(s) *

What language is the intervention/app in? If multiple languages are available, separate by comma (e.g. "English, French")

Vietnamese

URL of your Intervention Website or App

e.g. a direct link to the mobile app on app in appstore (itunes, Google Play), or URL of the website. If the intervention is a DVD or hardware, you can also link to an Amazon page.

Câu trả lời của bạn

URL of an image/screenshot (optional)

Câu trả lời của bạn

Accessibility *

Can an enduser access the intervention presently?

- ○ access is free and open
- ○ access only for special usergroups, not open
- ○ access is open to everyone, but requires payment/subscription/in-app purchases
- ◉ app/intervention no longer accessible
- ○ Mục khác:

Primary Medical Indication/Disease/Condition *

e.g. "Stress", "Diabetes", or define the target group in brackets after the condition, e.g. "Autism (Parents of children with)", "Alzheimers (Informal Caregivers of)"

HIV/AIDS patients

Primary Outcomes measured in trial *

comma-separated list of primary outcomes reported in the trial

Adherence, HIV Treatment Adherence Self-Effi

Secondary/other outcomes

Are there any other outcomes the intervention is expected to affect?

Câu trả lời của bạn

Recommended "Dose" *

What do the instructions for users say on how often the app should be used?

- (selected) Approximately Daily
- Approximately Weekly
- Approximately Monthly
- Approximately Yearly
- "as needed"
- Mục khác:

Approx. Percentage of Users (starters) still using the app as recommended after 3 months *

- (selected) unknown / not evaluated
- 0-10%
- 11-20%
- 21-30%
- 31-40%
- 41-50%
- 51-60%
- 61-70%
- 71%-80%
- 81-90%
- 91-100%
- Mục khác:

Overall, was the app/intervention effective? *

- (selected) yes: all primary outcomes were significantly better in intervention group vs control
- partly: SOME primary outcomes were significantly better in intervention group vs control
- no statistically significant difference between control and intervention
- potentially harmful: control was significantly better than intervention in one or more outcomes
- inconclusive: more research is needed
- Mục khác:

Article Preparation Status/Stage *

At which stage in your article preparation are you currently (at the time you fill in this form)

- not submitted yet - in early draft status
- not submitted yet - in late draft status, just before submission
- submitted to a journal but not reviewed yet
- submitted to a journal and after receiving initial reviewer comments
- (selected) submitted to a journal and accepted, but not published yet
- published
- Mục khác:

Journal *

If you already know where you will submit this paper (or if it is already submitted), please provide the journal name (if it is not JMIR, provide the journal name under "other")

- ○ not submitted yet / unclear where I will submit this
- ◉ Journal of Medical Internet Research (JMIR)
- ○ JMIR mHealth and UHealth
- ○ JMIR Serious Games
- ○ JMIR Mental Health
- ○ JMIR Public Health
- ○ JMIR Formative Research
- ○ Other JMIR sister journal
- ○ Mục khác:

Is this a full powered effectiveness trial or a pilot/feasibility trial? *

- ○ Pilot/feasibility
- ◉ Fully powered

Manuscript tracking number *

If this is a JMIR submission, please provide the manuscript tracking number under "other" (The ms tracking number can be found in the submission acknowledgement email, or when you login as author in JMIR. If the paper is already published in JMIR, then the ms tracking number is the four-digit number at the end of the DOI, to be found at the bottom of each published article in JMIR)

- ○ no ms number (yet) / not (yet) submitted to / published in JMIR
- ◉ Mục khác: ms#43432

TITLE AND ABSTRACT

1a) TITLE: Identification as a randomized trial in the title

1a) Does your paper address CONSORT item 1a? *

I.e does the title contain the phrase "Randomized Controlled Trial"? (if not, explain the reason under "other")

- ◉ yes
- ○ Mục khác:

1a-i) Identify the mode of delivery in the title

Identify the mode of delivery. Preferably use “web-based” and/or “mobile” and/or “electronic game” in the title. Avoid ambiguous terms like “online”, “virtual”, “interactive”. Use “Internet-based” only if Intervention includes non-web-based Internet components (e.g. email), use “computer-based” or “electronic” only if offline products are used. Use “virtual” only in the context of “virtual reality” (3-D worlds). Use “online” only in the context of “online support groups”. Complement or substitute product names with broader terms for the class of products (such as “mobile” or “smart phone” instead of “iphone”), especially if the application runs on different platforms.

	1	2	3	4	5	
subitem not at all important	○	○	○	●	○	essential

Xóa lựa chọn

Does your paper address subitem 1a-i? *

Copy and paste relevant sections from manuscript title (include quotes in quotation marks "like this" to indicate direct quotes from your manuscript), or elaborate on this item by providing additional information not in the ms, or briefly explain why the item is not applicable/relevant for your study

"Mobile Phone–Based Intervention"

1a-ii) Non-web-based components or important co-interventions in title

Mention non-web-based components or important co-interventions in title, if any (e.g., “with telephone support”).

	1	2	3	4	5	
subitem not at all important	○	○	●	○	○	essential

Xóa lựa chọn

Does your paper address subitem 1a-ii?

Copy and paste relevant sections from manuscript title (include quotes in quotation marks "like this" to indicate direct quotes from your manuscript), or elaborate on this item by providing additional information not in the ms, or briefly explain why the item is not applicable/relevant for your study

No other co-interventions added to title.

1a-iii) Primary condition or target group in the title

Mention primary condition or target group in the title, if any (e.g., "for children with Type I Diabetes") Example: A Web-based and Mobile Intervention with Telephone Support for Children with Type I Diabetes: Randomized Controlled Trial

	1	2	3	4	5	
subitem not at all important	○	○	◉	○	○	essential

Xóa lựa chọn

Does your paper address subitem 1a-iii? *

Copy and paste relevant sections from manuscript title (include quotes in quotation marks "like this" to indicate direct quotes from your manuscript), or elaborate on this item by providing additional information not in the ms, or briefly explain why the item is not applicable/relevant for your study

"Health Behaviors and HIV/AIDS Treatment Management"

1b) ABSTRACT: Structured summary of trial design, methods, results, and conclusions

NPT extension: Description of experimental treatment, comparator, care providers, centers, and blinding status.

1b-i) Key features/functionalities/components of the intervention and comparator in the METHODS section of the ABSTRACT

Mention key features/functionalities/components of the intervention and comparator in the abstract. If possible, also mention theories and principles used for designing the site. Keep in mind the needs of systematic reviewers and indexers by including important synonyms. (Note: Only report in the abstract what the main paper is reporting. If this information is missing from the main body of text, consider adding it)

	1	2	3	4	5	
subitem not at all important	○	○	○	◉	○	essential

Xóa lựa chọn

Does your paper address subitem 1b-i? *

Copy and paste relevant sections from the manuscript abstract (include quotes in quotation marks "like this" to indicate direct quotes from your manuscript), or elaborate on this item by providing additional information not in the ms, or briefly explain why the item is not applicable/relevant for your study

"Both the intervention group (243 patients) and the control group (187 patients) received regular consultations with doctors and then participated in 1-month and 3-month follow-up visits. Patients in the intervention group received a theory-driven smartphone app to facilitate medication adherence and self-efficacy in HIV patients. Measurements were developed based on the Health Belief Model, which included the Visual Analog Scale of ART Adherence, HIV Treatment Adherence Self-Efficacy Scale, and HIV Symptom Management Self-Efficacy Scale. We also included the Patients Health Questionnaire-9 (PHQ-9) to assess patients' mental health throughout treatment."

1b-ii) Level of human involvement in the METHODS section of the ABSTRACT

Clarify the level of human involvement in the abstract, e.g., use phrases like “fully automated” vs. “therapist/nurse/care provider/physician-assisted” (mention number and expertise of providers involved, if any). (Note: Only report in the abstract what the main paper is reporting. If this information is missing from the main body of text, consider adding it)

	1	2	3	4	5	
subitem not at all important	○	○	○	◉	○	essential

Xóa lựa chọn

Does your paper address subitem 1b-ii?

Copy and paste relevant sections from the manuscript abstract (include quotes in quotation marks "like this" to indicate direct quotes from your manuscript), or elaborate on this item by providing additional information not in the ms, or briefly explain why the item is not applicable/relevant for your study

"Both the intervention group (243 patients) and the control group (187 patients) received regular consultations with doctors and then participated in 1-month and 3-month follow-up visits. Patients in the intervention group received a theory-driven smartphone app to facilitate medication adherence and self-efficacy in HIV patients."

1b-iii) Open vs. closed, web-based (self-assessment) vs. face-to-face assessments in the METHODS section of the ABSTRACT

Mention how participants were recruited (online vs. offline), e.g., from an open access website or from a clinic or a closed online user group (closed usergroup trial), and clarify if this was a purely web-based trial, or there were face-to-face components (as part of the intervention or for assessment). Clearly say if outcomes were self-assessed through questionnaires (as common in web-based trials). Note: In traditional offline trials, an open trial (open-label trial) is a type of clinical trial in which both the researchers and participants know which treatment is being administered. To avoid confusion, use “blinded” or “unblinded” to indicated the level of blinding instead of “open”, as “open” in web-based trials usually refers to “open access” (i.e. participants can self-enrol). (Note: Only report in the abstract what the main paper is reporting. If this information is missing from the main body of text, consider adding it)

	1	2	3	4	5	
subitem not at all important	○	○	○	◉	○	essential

Xóa lựa chọn

Does your paper address subitem 1b-iii?

Copy and paste relevant sections from the manuscript abstract (include quotes in quotation marks "like this" to indicate direct quotes from your manuscript), or elaborate on this item by providing additional information not in the ms, or briefly explain why the item is not applicable/relevant for your study

"We performed a randomized controlled trial on 428 HIV patients in two of the largest HIV clinics in Hanoi, Vietnam. Both the intervention group (243 patients) and the control group (187 patients) received regular consultations with doctors and then participated in 1-month and 3-month follow-up visits. Patients in the intervention group received a theory-driven smartphone app to facilitate medication adherence and self-efficacy in HIV patients. Measurements were developed based on the Health Belief Model, which included the Visual Analog Scale of ART Adherence, HIV Treatment Adherence Self-Efficacy Scale, and HIV Symptom Management Self-Efficacy Scale. We also included the Patients Health Questionnaire-9 (PHQ-9) to assess patients’ mental health throughout treatment."

1b-iv) RESULTS section in abstract must contain use data

Report number of participants enrolled/assessed in each group, the use/uptake of the intervention (e.g., attrition/adherence metrics, use over time, number of logins etc.), in addition to primary/secondary outcomes. (Note: Only report in the abstract what the main paper is reporting. If this information is missing from the main body of text, consider adding it)

	1	2	3	4	5	
subitem not at all important	○	○	◉	○	○	essential

Xóa lựa chọn

Does your paper address subitem 1b-iv?

Copy and paste relevant sections from the manuscript abstract (include quotes in quotation marks "like this" to indicate direct quotes from your manuscript), or elaborate on this item by providing additional information not in the ms, or briefly explain why the item is not applicable/relevant for your study

The number of participants enrolled/assessed in each group were presented in Methods section in Abstract.

1b-v) CONCLUSIONS/DISCUSSION in abstract for negative trials

Conclusions/Discussions in abstract for negative trials: Discuss the primary outcome - if the trial is negative (primary outcome not changed), and the intervention was not used, discuss whether negative results are attributable to lack of uptake and discuss reasons. (Note: Only report in the abstract what the main paper is reporting. If this information is missing from the main body of text, consider adding it)

	1	2	3	4	5	
subitem not at all important	○	○	◉	○	○	essential

Xóa lựa chọn

Does your paper address subitem 1b-v?

Copy and paste relevant sections from the manuscript abstract (include quotes in quotation marks "like this" to indicate direct quotes from your manuscript), or elaborate on this item by providing additional information not in the ms, or briefly explain why the item is not applicable/relevant for your study

"Our study demonstrated that the mHealth app could improve the overall ART adherence self-efficacy of patients. Further studies with larger sample sizes and longer follow-up periods are needed to support our findings."

INTRODUCTION

2a) In INTRODUCTION: Scientific background and explanation of rationale

2a-i) Problem and the type of system/solution

Describe the problem and the type of system/solution that is object of the study: intended as stand-alone intervention vs. incorporated in broader health care program? Intended for a particular patient population? Goals of the intervention, e.g., being more cost-effective to other interventions, replace or complement other solutions? (Note: Details about the intervention are provided in “Methods” under 5)

	1	2	3	4	5	
subitem not at all important	○	○	○	●	○	essential

Xóa lựa chọn

Does your paper address subitem 2a-i? *

Copy and paste relevant sections from the manuscript (include quotes in quotation marks "like this" to indicate direct quotes from your manuscript), or elaborate on this item by providing additional information not in the ms, or briefly explain why the item is not applicable/relevant for your study

"For decades, HIV/AIDS has been regarded as one of the most serious public health crises in history"
"Recent advances in eHealth and mobile health (mHealth) technologies have transformed health care approaches, especially in the delivery of HIV/AIDS treatments"
"the aim of this study was to evaluate the feasibility and efficacy of a theory-based mHealth intervention on HIV treatment adherence, self-efficacy, and health behaviors among people living with HIV in Vietnam"

2a-ii) Scientific background, rationale: What is known about the (type of) system

Scientific background, rationale: What is known about the (type of) system that is the object of the study (be sure to discuss the use of similar systems for other conditions/diagnoses, if appropiate), motivation for the study, i.e. what are the reasons for and what is the context for this specific study, from which stakeholder viewpoint is the study performed, potential impact of findings [2]. Briefly justify the choice of the comparator.

	1	2	3	4	5	
subitem not at all important	○	○	○	●	○	essential

Xóa lựa chọn

Does your paper address subitem 2a-ii? *

Copy and paste relevant sections from the manuscript (include quotes in quotation marks "like this" to indicate direct quotes from your manuscript), or elaborate on this item by providing additional information not in the ms, or briefly explain why the item is not applicable/relevant for your study

"Recent advances in eHealth and mobile health (mHealth) technologies have transformed health care approaches, especially in the delivery of HIV/AIDS treatments"
"Behavioral change theories should be incorporated in the design of mHealth interventions with considerations of accessibility and cost-effectiveness. It is important to understand the core mechanism underlying an intervention for effective transition to technological platforms."
"Our study was developed on the Health Belief Model, where perceived severity and self-efficacy were measured throughout the study period and follow-up meetings for comparison, and personalized messages were sent as cues to actions"

2b) In INTRODUCTION: Specific objectives or hypotheses

Does your paper address CONSORT subitem 2b? *

Copy and paste relevant sections from the manuscript (include quotes in quotation marks "like this" to indicate direct quotes from your manuscript), or elaborate on this item by providing additional information not in the ms, or briefly explain why the item is not applicable/relevant for your study

"Our study was developed on the Health Belief Model, where perceived severity and self-efficacy were measured throughout the study period and follow-up meetings for comparison, and personalized messages were sent as cues to actions"
"the aim of this study was to evaluate the feasibility and efficacy of a theory-based mHealth intervention on HIV treatment adherence, self-efficacy, and health behaviors among people living with HIV in Vietnam."

METHODS

3a) Description of trial design (such as parallel, factorial) including allocation ratio

Does your paper address CONSORT subitem 3a? *

Copy and paste relevant sections from the manuscript (include quotes in quotation marks "like this" to indicate direct quotes from your manuscript), or elaborate on this item by providing additional information not in the ms, or briefly explain why the item is not applicable/relevant for your study

"Convenience sampling was used in the selection of participants. All participants agreed to participate in this study by signing a written informed consent form. By the end of the sample recruitment period, a total of 495 patients were recruited and a baseline assessment was performed [16,17]. Participants were then randomized into the intervention arm (n=248) and the control arm (n=247) by a computer software. After the randomization phase, 65 participants were excluded, including 5 from the intervention arm and 60 from the control arm with the following reasons: family issues (n=2), transferred to other clinics (n=3), used other HIV-assist smartphone apps (n=55), stopped participating in the study (n=2), and technical problems with the smartphone (n=4). A total of 428 HIV patients fulfilled the inclusion criteria and participated in the intervention phases, including 243 patients in the intervention arm and 187 patients in the control arm. Five patients in the intervention group did not complete regular follow-ups after 1 month of the intervention and were excluded. Finally, data of 238 patients in the intervention arm and 187 patients in the control arm were analyzed."

3b) Important changes to methods after trial commencement (such as eligibility criteria), with reasons

Does your paper address CONSORT subitem 3b? *

Copy and paste relevant sections from the manuscript (include quotes in quotation marks "like this" to indicate direct quotes from your manuscript), or elaborate on this item by providing additional information not in the ms, or briefly explain why the item is not applicable/relevant for your study

"After the randomization phase, 65 participants were excluded, including 5 from the intervention arm and 60 from the control arm with the following reasons: family issues (n=2), transferred to other clinics (n=3), used other HIV-assist smartphone apps (n=55), stopped participating in the study (n=2), and technical problems with the smartphone (n=4). A total of 428 HIV patients fulfilled the inclusion criteria and participated in the intervention phases, including 243 patients in the intervention arm and 187 patients in the control arm. Five patients in the intervention group did not complete regular follow-ups after 1 month of the intervention and were excluded."

3b-i) Bug fixes, Downtimes, Content Changes

Bug fixes, Downtimes, Content Changes: ehealth systems are often dynamic systems. A description of changes to methods therefore also includes important changes made on the intervention or comparator during the trial (e.g., major bug fixes or changes in the functionality or content) (5-iii) and other “unexpected events” that may have influenced study design such as staff changes, system failures/downtimes, etc. [2].

	1	2	3	4	5	
subitem not at all important	○	○	○	○	○	essential

Does your paper address subitem 3b-i?

Copy and paste relevant sections from the manuscript (include quotes in quotation marks "like this" to indicate direct quotes from your manuscript), or elaborate on this item by providing additional information not in the ms, or briefly explain why the item is not applicable/relevant for your study

No bug fixes, Downtimes or Content fixes are recorded or noted.

4a) Eligibility criteria for participants

Does your paper address CONSORT subitem 4a? *

Copy and paste relevant sections from the manuscript (include quotes in quotation marks "like this" to indicate direct quotes from your manuscript), or elaborate on this item by providing additional information not in the ms, or briefly explain why the item is not applicable/relevant for your study

"This was a randomized controlled trial including participants recruited from outpatient HIV clinics at Bach Mai Hospital and Ha Dong General Hospital from March 2018 to December 2019."
"Patients were excluded if they were using any other HIV-assist smartphone app at the time of the study, or had cognitive impairments or disabilities that would hinder the use of our app."

4a-i) Computer / Internet literacy

Computer / Internet literacy is often an implicit “de facto” eligibility criterion - this should be explicitly clarified.

	1	2	3	4	5	
subitem not at all important	○	○	◉	○	○	essential

Xóa lựa chọn

Does your paper address subitem 4a-i?

Copy and paste relevant sections from the manuscript (include quotes in quotation marks "like this" to indicate direct quotes from your manuscript), or elaborate on this item by providing additional information not in the ms, or briefly explain why the item is not applicable/relevant for your study

Computer/Internet literacy was not specifically required for eligibility to the trial.

4a-ii) Open vs. closed, web-based vs. face-to-face assessments:

Open vs. closed, web-based vs. face-to-face assessments: Mention how participants were recruited (online vs. offline), e.g., from an open access website or from a clinic, and clarify if this was a purely web-based trial, or there were face-to-face components (as part of the intervention or for assessment), i.e., to what degree got the study team to know the participant. In online-only trials, clarify if participants were quasi-anonymous and whether having multiple identities was possible or whether technical or logistical measures (e.g., cookies, email confirmation, phone calls) were used to detect/prevent these.

	1	2	3	4	5	
subitem not at all important	○	○	○	◉	○	essential

Xóa lựa chọn

Does your paper address subitem 4a-ii? *

Copy and paste relevant sections from the manuscript (include quotes in quotation marks "like this" to indicate direct quotes from your manuscript), or elaborate on this item by providing additional information not in the ms, or briefly explain why the item is not applicable/relevant for your study

"This was a randomized controlled trial including participants recruited from outpatient HIV clinics at Bach Mai Hospital and Ha Dong General Hospital from March 2018 to December 2019. "
"Patients were excluded if they were using any other HIV-assist smartphone app at the time of the study, or had cognitive impairments or disabilities that would hinder the use of our app. Convenience sampling was used in the selection of participants."

4a-iii) Information giving during recruitment

Information given during recruitment. Specify how participants were briefed for recruitment and in the informed consent procedures (e.g., publish the informed consent documentation as appendix, see also item X26), as this information may have an effect on user self-selection, user expectation and may also bias results.

	1	2	3	4	5	
subitem not at all important	○	○	○	◉	○	essential

Xóa lựa chọn

Does your paper address subitem 4a-iii?

Copy and paste relevant sections from the manuscript (include quotes in quotation marks "like this" to indicate direct quotes from your manuscript), or elaborate on this item by providing additional information not in the ms, or briefly explain why the item is not applicable/relevant for your study

"All participants agreed to participate in this study by signing a written informed consent form."
"Participants in both the intervention and control groups received regular consultations from doctors at the clinic per Vietnam's guidelines for HIV/AIDS patient care. Patients in the intervention group received a theory-based smartphone app (eCARE app) for promoting medication adherence and self-management in HIV patients. Participants undertook the assigned intervention immediately after performing the baseline "

4b) Settings and locations where the data were collected

Does your paper address CONSORT subitem 4b? *

Copy and paste relevant sections from the manuscript (include quotes in quotation marks "like this" to indicate direct quotes from your manuscript), or elaborate on this item by providing additional information not in the ms, or briefly explain why the item is not applicable/relevant for your study

"This was a randomized controlled trial including participants recruited from outpatient HIV clinics at Bach Mai Hospital and Ha Dong General Hospital from March 2018 to December 2019. These are among the most popular hospitals for HIV treatment in Hanoi, Vietnam, and are located in different parts of Hanoi to ensure geographical variety and different levels of administration. The Bach Mai HIV Outpatient Clinic (a central-special hospital) is funded by the government to provide ART to nearly 3000 HIV-positive patients in Hanoi, while the HIV Clinic in Ha Dong General Hospital (provincial-level hospital) provides insurance-covered testing and treatment as well as free counseling."

4b-i) Report if outcomes were (self-)assessed through online questionnaires

Clearly report if outcomes were (self-)assessed through online questionnaires (as common in web-based trials) or otherwise.

	1	2	3	4	5	
subitem not at all important	○	○	○	○	○	essential

Does your paper address subitem 4b-i? *

Copy and paste relevant sections from the manuscript (include quotes in quotation marks "like this" to indicate direct quotes from your manuscript), or elaborate on this item by providing additional information not in the ms, or briefly explain why the item is not applicable/relevant for your study

Our study used face-to-face interview.

4b-ii) Report how institutional affiliations are displayed

Report how institutional affiliations are displayed to potential participants [on ehealth media], as affiliations with prestigious hospitals or universities may affect volunteer rates, use, and reactions with regards to an intervention.(Not a required item – describe only if this may bias results)

	1	2	3	4	5	
subitem not at all important	○	○	◉	○	○	essential

Xóa lựa chọn

Does your paper address subitem 4b-ii?

Copy and paste relevant sections from the manuscript (include quotes in quotation marks "like this" to indicate direct quotes from your manuscript), or elaborate on this item by providing additional information not in the ms, or briefly explain why the item is not applicable/relevant for your study

Institutional affiliations were not displayed on recruitment materials.

5) The interventions for each group with sufficient details to allow replication, including how and when they were actually administered

5-i) Mention names, credential, affiliations of the developers, sponsors, and owners

Mention names, credential, affiliations of the developers, sponsors, and owners [6] (if authors/evaluators are owners or developer of the software, this needs to be declared in a “Conflict of interest” section or mentioned elsewhere in the manuscript).

	1	2	3	4	5	
subitem not at all important	○	○	●	○	○	essential

Xóa lựa chọn

Does your paper address subitem 5-i?

Copy and paste relevant sections from the manuscript (include quotes in quotation marks "like this" to indicate direct quotes from your manuscript), or elaborate on this item by providing additional information not in the ms, or briefly explain why the item is not applicable/relevant for your study

"Mention names, credential, affiliations of the developers, sponsors, and owners
Mention names, credential, affiliations of the developers, sponsors, and owners [6] (if authors/evaluators are owners or developer of the software, this needs to be declared in a “Conflict of interest” section or mentioned elsewhere in the manuscript)."

5-ii) Describe the history/development process

Describe the history/development process of the application and previous formative evaluations (e.g., focus groups, usability testing), as these will have an impact on adoption/use rates and help with interpreting results.

	1	2	3	4	5	
subitem not at all important	○	○	●	○	○	essential

Xóa lựa chọn

Does your paper address subitem 5-ii?

Copy and paste relevant sections from the manuscript (include quotes in quotation marks "like this" to indicate direct quotes from your manuscript), or elaborate on this item by providing additional information not in the ms, or briefly explain why the item is not applicable/relevant for your study

"The first version was designed as a web-based simulator and evaluated by experts in the field of HIV/AIDS treatment as well as by some patients in the study population. After fixing the bugs, the final version (in terms of content) was developed and put into beta form. The testing phase included stability testing on iOS and Android platforms and testing the effectiveness of the app. The trial phase was conducted on 20 patients to investigate their requirements for the app in terms of form and content. The intervention group was given access to the eCARE app through medical staff at the treatment facility."

5-iii) Revisions and updating

Revisions and updating. Clearly mention the date and/or version number of the application/intervention (and comparator, if applicable) evaluated, or describe whether the intervention underwent major changes during the evaluation process, or whether the development and/or content was "frozen" during the trial. Describe dynamic components such as news feeds or changing content which may have an impact on the replicability of the intervention (for unexpected events see item 3b).

	1	2	3	4	5	
subitem not at all important	○	○	◉	○	○	essential

Xóa lựa chọn

Does your paper address subitem 5-iii?

Copy and paste relevant sections from the manuscript (include quotes in quotation marks "like this" to indicate direct quotes from your manuscript), or elaborate on this item by providing additional information not in the ms, or briefly explain why the item is not applicable/relevant for your study

No major changes occurred to the intervention during the study.

5-iv) Quality assurance methods

Provide information on quality assurance methods to ensure accuracy and quality of information provided [1], if applicable.

	1	2	3	4	5	
subitem not at all important	○	○	●	○	○	essential

Xóa lựa chọn

Does your paper address subitem 5-iv?

Copy and paste relevant sections from the manuscript (include quotes in quotation marks "like this" to indicate direct quotes from your manuscript), or elaborate on this item by providing additional information not in the ms, or briefly explain why the item is not applicable/relevant for your study

"To handle missing data, we used the listwise deletion method to clean the data before analysis"

5-v) Ensure replicability by publishing the source code, and/or providing screenshots/screen-capture video, and/or providing flowcharts of the algorithms used

Ensure replicability by publishing the source code, and/or providing screenshots/screen-capture video, and/or providing flowcharts of the algorithms used. Replicability (i.e., other researchers should in principle be able to replicate the study) is a hallmark of scientific reporting.

	1	2	3	4	5	
subitem not at all important	○	○	○	◉	○	essential

Xóa lựa chọn

Does your paper address subitem 5-v?

Copy and paste relevant sections from the manuscript (include quotes in quotation marks "like this" to indicate direct quotes from your manuscript), or elaborate on this item by providing additional information not in the ms, or briefly explain why the item is not applicable/relevant for your study

Flowchart of the study was prensented in Figure 1.

5-vi) Digital preservation

Digital preservation: Provide the URL of the application, but as the intervention is likely to change or disappear over the course of the years; also make sure the intervention is archived (Internet Archive, webcitation.org, and/or publishing the source code or screenshots/videos alongside the article). As pages behind login screens cannot be archived, consider creating demo pages which are accessible without login.

	1	2	3	4	5	
subitem not at all important	○	○	◉	○	○	essential

Xóa lựa chọn

Does your paper address subitem 5-vi?

Copy and paste relevant sections from the manuscript (include quotes in quotation marks "like this" to indicate direct quotes from your manuscript), or elaborate on this item by providing additional information not in the ms, or briefly explain why the item is not applicable/relevant for your study

This intervention is not currently available. The screen shot of the intervention is shown in Figure 2.

5-vii) Access

Access: Describe how participants accessed the application, in what setting/context, if they had to pay (or were paid) or not, whether they had to be a member of specific group. If known, describe how participants obtained “access to the platform and Internet” [1]. To ensure access for editors/reviewers/readers, consider to provide a “backdoor” login account or demo mode for reviewers/readers to explore the application (also important for archiving purposes, see vi).

	1	2	3	4	5	
subitem not at all important	○	○	○	◉	○	essential

Xóa lựa chọn

Does your paper address subitem 5-vii? *

Copy and paste relevant sections from the manuscript (include quotes in quotation marks "like this" to indicate direct quotes from your manuscript), or elaborate on this item by providing additional information not in the ms, or briefly explain why the item is not applicable/relevant for your study

"Participants in both the intervention and control groups received regular consultations from doctors at the clinic per Vietnam’s guidelines for HIV/AIDS patient care. Patients in the intervention group received a theory-based smartphone app (eCARE app) for promoting medication adherence and self-management in HIV patients. Participants undertook the assigned intervention immediately after performing the baseline survey."

5-viii) Mode of delivery, features/functionalities/components of the intervention and comparator, and the theoretical framework

Describe mode of delivery, features/functionalities/components of the intervention and comparator, and the theoretical framework [6] used to design them (instructional strategy [1], behaviour change techniques, persuasive features, etc., see e.g., [7, 8] for terminology). This includes an in-depth description of the content (including where it is coming from and who developed it) [1],” whether [and how] it is tailored to individual circumstances and allows users to track their progress and receive feedback” [6]. This also includes a description of communication delivery channels and – if computer-mediated communication is a component – whether communication was synchronous or asynchronous [6]. It also includes information on presentation strategies [1], including page design principles, average amount of text on pages, presence of hyperlinks to other resources, etc. [1].

	1	2	3	4	5	
subitem not at all important	○	○	○	○	◉	essential

Xóa lựa chọn

Does your paper address subitem 5-viii? *

Copy and paste relevant sections from the manuscript (include quotes in quotation marks "like this" to indicate direct quotes from your manuscript), or elaborate on this item by providing additional information not in the ms, or briefly explain why the item is not applicable/relevant for your study

"The eCARE app was developed based on the Analysis, Design, Development, Implementation, and Evaluation (ADDIE) model. We first performed a literature review to build the content components for the app. The messages of the app were developed based on Social Cognitive Theory, the Health Belief Model, and the Integrated Theory of Behavior Change."
"Participants in both the intervention and control groups received regular consultations from doctors at the clinic per Vietnam’s guidelines for HIV/AIDS patient care. Patients in the intervention group received a theory-based smartphone app (eCARE app) for promoting medication adherence and self-management in HIV patients. Participants undertook the assigned intervention immediately after performing the baseline survey."

5-ix) Describe use parameters

Describe use parameters (e.g., intended “doses” and optimal timing for use). Clarify what instructions or recommendations were given to the user, e.g., regarding timing, frequency, heaviness of use, if any, or was the intervention used ad libitum.

	1	2	3	4	5	
subitem not at all important	○	○	○	○	◉	essential

Xóa lựa chọn

Does your paper address subitem 5-ix?

Copy and paste relevant sections from the manuscript (include quotes in quotation marks "like this" to indicate direct quotes from your manuscript), or elaborate on this item by providing additional information not in the ms, or briefly explain why the item is not applicable/relevant for your study

"The final smartphone app (eCARE) contains the following core functions: (1) personal medical record; (2) medication reminder; (3) behavior monitoring (including tobacco smoking, alcohol drinking, and illegal substance use); (4) connection to health facilities; (5) guidelines, information, and news; and (6) contact to HIV clinics. Figure 2 illustrates the main functions of the app.
Personal Medical Record
This section retrieves and consolidates all the information from the respective medical databases to create a centralized medical record for the patient. This approach can avoid missing information that might hinder future health care interventions. Patients are provided with an overview of their medical information and their treatment plans.
Medication Reminder
The medication reminder provides simple daily reminders to take medication, which ultimately improves adherence to ART. The reminders are set as alarms or sent as text messages at designated times. This function also allows health care staff to track and monitor patients' compliance.
Behavior Monitoring (Tobacco Smoking, Alcohol Drinking, and Illegal Substance Use)
The behavior monitoring functionality allows individuals to self-report their behaviors daily. The app disseminates a variety of messages (through phone messages, app notifications, and videos) to help individuals change their unhealthy behaviors. These messages are developed based on the Social Cognitive Theory and the Health Belief Model. Each message is also individualized according to the baseline information of the patient such as gender, age, location, smoking, alcohol drinking, substance use, health status, and medication adherence (eg, "Let yourself and your loved ones be proud that you have quit smoking for a healthy life" or "Adhering medication is the only way to prevent HIV from progressing").
Guidelines, Information, and News
News and guidelines about HIV/AIDS treatment and relevant issues are provided within the app and are updated on a daily basis.
Contact to HIV Clinics
This function allows users to interact directly with medical staff when they need health advice. In emergencies, individuals will be connected to the nearest medical facility."

5-x) Clarify the level of human involvement

Clarify the level of human involvement (care providers or health professionals, also technical assistance) in the e-intervention or as co-intervention (detail number and expertise of professionals involved, if any, as well as “type of assistance offered, the timing and frequency of the support, how it is initiated, and the medium by which the assistance is delivered”. It may be necessary to distinguish between the level of human involvement required for the trial, and the level of human involvement required for a routine application outside of a RCT setting (discuss under item 21 – generalizability).

	1	2	3	4	5	
subitem not at all important	○	○	○	●	○	essential

Xóa lựa chọn

Does your paper address subitem 5-x?

Copy and paste relevant sections from the manuscript (include quotes in quotation marks "like this" to indicate direct quotes from your manuscript), or elaborate on this item by providing additional information not in the ms, or briefly explain why the item is not applicable/relevant for your study

"Contact to HIV Clinics
This function allows users to interact directly with medical staff when they need health advice. In emergencies, individuals will be connected to the nearest medical facility."
"Trained staff members assisted in data collection."

5-xi) Report any prompts/reminders used

Report any prompts/reminders used: Clarify if there were prompts (letters, emails, phone calls, SMS) to use the application, what triggered them, frequency etc. It may be necessary to distinguish between the level of prompts/reminders required for the trial, and the level of prompts/reminders for a routine application outside of a RCT setting (discuss under item 21 – generalizability).

	1	2	3	4	5	
subitem not at all important	○	○	○	◉	○	essential

Xóa lựa chọn

Does your paper address subitem 5-xi? *

Copy and paste relevant sections from the manuscript (include quotes in quotation marks "like this" to indicate direct quotes from your manuscript), or elaborate on this item by providing additional information not in the ms, or briefly explain why the item is not applicable/relevant for your study

"Medication Reminder
The medication reminder provides simple daily reminders to take medication, which ultimately improves adherence to ART. The reminders are set as alarms or sent as text messages at designated times. This function also allows health care staff to track and monitor patients' compliance."

5-xii) Describe any co-interventions (incl. training/support)

Describe any co-interventions (incl. training/support): Clearly state any interventions that are provided in addition to the targeted eHealth intervention, as ehealth intervention may not be designed as stand-alone intervention. This includes training sessions and support [1]. It may be necessary to distinguish between the level of training required for the trial, and the level of training for a routine application outside of a RCT setting (discuss under item 21 – generalizability.

	1	2	3	4	5	
subitem not at all important	○	○	◉	○	○	essential

Xóa lựa chọn

Does your paper address subitem 5-xii? *

Copy and paste relevant sections from the manuscript (include quotes in quotation marks "like this" to indicate direct quotes from your manuscript), or elaborate on this item by providing additional information not in the ms, or briefly explain why the item is not applicable/relevant for your study

"Participants in both the intervention and control groups received regular consultations from doctors at the clinic per Vietnam’s guidelines for HIV/AIDS patient care'

6a) Completely defined pre-specified primary and secondary outcome measures, including how and when they were assessed

Does your paper address CONSORT subitem 6a? *

Copy and paste relevant sections from the manuscript (include quotes in quotation marks "like this" to indicate direct quotes from your manuscript), or elaborate on this item by providing additional information not in the ms, or briefly explain why the item is not applicable/relevant for your study

"At the 3-month follow-up, they were further asked “Compared to before participating in the study, how do you assess your drinking/smoking/drug use now?” with three answer options (increase, constant, decrease) to explore the change of participant’s risk behaviors.
The VAS of ART Adherence is a self-reported scale that was used to assess adherence to ART treatment among participants"
"The HIV-ASES includes 12 items and is used to assess patients’ confidence in adherence to treatment plans, including medications, diet, exercise, and the consumption of vitamins."
"The HIV Symptom Management Self-Efficacy Scale has a total of 10 items adapted from the abbreviated 6-item Chronic Disease Self-Efficacy Scale. The scale evaluates four domains, including symptom control, role function, emotional function, and communication with physicians. This scale measures the confidence in patients’ capacity to manage HIV symptoms."
"Although these two scales, the HIV-ASES and the HIV Symptom Management Self-Efficacy Scale, are important measures to assess patients’ readiness and capacity to initiate treatment, after the pilot trial, we found that these scales were not able to accurately reflect the change in the first month of ART among patients at Bach Mai Hospital, resulting in a very high ceiling effect with almost all patients showing the highest scores. Thus, we decided not to apply these scales in the 1-month follow-up. The reason was that Bach Mai Hospital is the largest, special-level hospital in Vietnam; thus, the procedure for initiating treatment was implemented very comprehensively. The patients’ readiness was improved substantially by various interventions, including family, group, and individual counseling; peer support; health checkups; and Opportunistic Infection (OI) treatment."

6a-i) Online questionnaires: describe if they were validated for online use and apply CHERRIES items to describe how the questionnaires were designed/deployed

If outcomes were obtained through online questionnaires, describe if they were validated for online use and apply CHERRIES items to describe how the questionnaires were designed/deployed [9].

	1	2	3	4	5	
subitem not at all important	○	○	◉	○	○	essential

Xóa lựa chọn

Does your paper address subitem 6a-i?

Copy and paste relevant sections from manuscript text

Our study used face-to-face interview.

6a-ii) Describe whether and how “use” (including intensity of use/dosage) was defined/measured/monitored

Describe whether and how “use” (including intensity of use/dosage) was defined/measured/monitored (logins, logfile analysis, etc.). Use/adoption metrics are important process outcomes that should be reported in any ehealth trial.

	1	2	3	4	5	
subitem not at all important	○	○	○	◉	○	essential

Xóa lựa chọn

Does your paper address subitem 6a-ii?

Copy and paste relevant sections from manuscript text

Paricipant engagement with intervention was measured through in-app metrics.

6a-iii) Describe whether, how, and when qualitative feedback from participants was obtained

Describe whether, how, and when qualitative feedback from participants was obtained (e.g., through emails, feedback forms, interviews, focus groups).

	1	2	3	4	5	
subitem not at all important	○	○	●	○	○	essential

Xóa lựa chọn

Does your paper address subitem 6a-iii?

Copy and paste relevant sections from manuscript text

Not applicable, no qualitative feedback was captured.

6b) Any changes to trial outcomes after the trial commenced, with reasons

Does your paper address CONSORT subitem 6b? *

Copy and paste relevant sections from the manuscript (include quotes in quotation marks "like this" to indicate direct quotes from your manuscript), or elaborate on this item by providing additional information not in the ms, or briefly explain why the item is not applicable/relevant for your study

"Although these two scales, the HIV-ASES and the HIV Symptom Management Self-Efficacy Scale, are important measures to assess patients' readiness and capacity to initiate treatment, after the pilot trial, we found that these scales were not able to accurately reflect the change in the first month of ART among patients at Bach Mai Hospital, resulting in a very high ceiling effect with almost all patients showing the highest scores. Thus, we decided not to apply these scales in the 1-month follow-up. The reason was that Bach Mai Hospital is the largest, special-level hospital in Vietnam; thus, the procedure for initiating treatment was implemented very comprehensively. The patients' readiness was improved substantially by various interventions, including family, group, and individual counseling; peer support; health checkups; and Opportunistic Infection (OI) treatment."

7a) How sample size was determined

NPT: When applicable, details of whether and how the clustering by care provides or centers was addressed

7a-i) Describe whether and how expected attrition was taken into account when calculating the sample size

Describe whether and how expected attrition was taken into account when calculating the sample size.

	1	2	3	4	5	
subitem not at all important	○	○	◉	○	○	essential

Xóa lựa chọn

Does your paper address subitem 7a-i?

Copy and paste relevant sections from manuscript title (include quotes in quotation marks "like this" to indicate direct quotes from your manuscript), or elaborate on this item by providing additional information not in the ms, or briefly explain why the item is not applicable/relevant for your study

"Convenience sampling was used in the selection of participants."

7b) When applicable, explanation of any interim analyses and stopping guidelines

Does your paper address CONSORT subitem 7b? *

Copy and paste relevant sections from the manuscript (include quotes in quotation marks "like this" to indicate direct quotes from your manuscript), or elaborate on this item by providing additional information not in the ms, or briefly explain why the item is not applicable/relevant for your study

Not applicable. Explanation of any interim analyses and stopping guidelines were provided from doctors at the clinic when patients return to received regular consultations.

8a) Method used to generate the random allocation sequence
NPT: When applicable, how care providers were allocated to each trial group

Does your paper address CONSORT subitem 8a? *

Copy and paste relevant sections from the manuscript (include quotes in quotation marks "like this" to indicate direct quotes from your manuscript), or elaborate on this item by providing additional information not in the ms, or briefly explain why the item is not applicable/relevant for your study

"data of 238 patients in the intervention arm and 187 patients in the control arm were analyzed."

8b) Type of randomisation; details of any restriction (such as blocking and block size)

Does your paper address CONSORT subitem 8b? *

Copy and paste relevant sections from the manuscript (include quotes in quotation marks "like this" to indicate direct quotes from your manuscript), or elaborate on this item by providing additional information not in the ms, or briefly explain why the item is not applicable/relevant for your study

Participants were randomized 1:1 (treatment:control). But after the randomization phase, 65 participants were excluded, including 5 from the intervention arm and 60 from the control arm with the following reasons: family issues (n=2), transferred to other clinics (n=3), used other HIV-assist smartphone apps (n=55), stopped participating in the study (n=2), and technical problems with the smartphone (n=4). A total of 428 HIV patients fulfilled the inclusion criteria and participated in the intervention phases, including 243 patients in the intervention arm and 187 patients in the control arm. Five patients in the intervention group did not complete regular follow-ups after 1 month of the intervention and were excluded. Finally, data of 238 patients in the intervention arm and 187 patients in the control arm were analyzed.

9) Mechanism used to implement the random allocation sequence (such as sequentially numbered containers), describing any steps taken to conceal the sequence until interventions were assigned

Does your paper address CONSORT subitem 9? *

Copy and paste relevant sections from the manuscript (include quotes in quotation marks "like this" to indicate direct quotes from your manuscript), or elaborate on this item by providing additional information not in the ms, or briefly explain why the item is not applicable/relevant for your study

Researcher randomizing participants were blond allocation until they had performed the randomization.

10) Who generated the random allocation sequence, who enrolled participants, and who assigned participants to interventions

Does your paper address CONSORT subitem 10? *

Copy and paste relevant sections from the manuscript (include quotes in quotation marks "like this" to indicate direct quotes from your manuscript), or elaborate on this item by providing additional information not in the ms, or briefly explain why the item is not applicable/relevant for your study

Researcher randomizing participants were blond allocation until they had performed the randomization. Random allocation sequence was generated by the management system. Participants were randomly allocated to either the control or treatment intervention.

11a) If done, who was blinded after assignment to interventions (for example, participants, care providers, those assessing outcomes) and how

NPT: Whether or not administering co-interventions were blinded to group assignment

11a-i) Specify who was blinded, and who wasn't

Specify who was blinded, and who wasn't. Usually, in web-based trials it is not possible to blind the participants [1, 3] (this should be clearly acknowledged), but it may be possible to blind outcome assessors, those doing data analysis or those administering co-interventions (if any).

	1	2	3	4	5	
subitem not at all important	○	○	○	○	○	essential

Does your paper address subitem 11a-i? *

Copy and paste relevant sections from the manuscript (include quotes in quotation marks "like this" to indicate direct quotes from your manuscript), or elaborate on this item by providing additional information not in the ms, or briefly explain why the item is not applicable/relevant for your study

Participants were blinded to the treatment allocation. The study was single blinded experimental design as researcher were aware of intervention allocation.

11a-ii) Discuss e.g., whether participants knew which intervention was the “intervention of interest” and which one was the “comparator”

Informed consent procedures (4a-ii) can create biases and certain expectations - discuss e.g., whether participants knew which intervention was the “intervention of interest” and which one was the “comparator”.

	1	2	3	4	5	
subitem not at all important	○	○	○	○	○	essential

Does your paper address subitem 11a-ii?

Copy and paste relevant sections from the manuscript (include quotes in quotation marks "like this" to indicate direct quotes from your manuscript), or elaborate on this item by providing additional information not in the ms, or briefly explain why the item is not applicable/relevant for your study

Pariticpants were not aware of the intervention of interest. No mention of a digital intervention was mentioned, see methods section for more detail

11b) If relevant, description of the similarity of interventions

(this item is usually not relevant for ehealth trials as it refers to similarity of a placebo or sham intervention to a active medication/intervention)

Does your paper address CONSORT subitem 11b? *

Copy and paste relevant sections from the manuscript (include quotes in quotation marks "like this" to indicate direct quotes from your manuscript), or elaborate on this item by providing additional information not in the ms, or briefly explain why the item is not applicable/relevant for your study

Not applicable. Pariticpants were not aware of the intervention of interest. No mention of a digital intervention was mentioned.

12a) Statistical methods used to compare groups for primary and secondary outcomes

NPT: When applicable, details of whether and how the clustering by care providers or centers was addressed

Does your paper address CONSORT subitem 12a? *

Copy and paste relevant sections from the manuscript (include quotes in quotation marks "like this" to indicate direct quotes from your manuscript), or elaborate on this item by providing additional information not in the ms, or briefly explain why the item is not applicable/relevant for your study

"For comparison of the change in patients' health behaviors and subgroups, we used the Wilcoxon rank-sum test; the Kruskal-Wallis test was used for comparisons of continuous variables and the $\chi 2$ test was used for comparisons of nominal variables. To determine the effect of the intervention, we tested the primary hypothesis that participants in the intervention group would have higher VAS of ART Adherence, HIV-ASES, and HIV Symptom Management Self-Efficacy Scale scores when compared to those of patients in the control group from baseline to follow-up at 1 and 3 months."

12a-i) Imputation techniques to deal with attrition / missing values

Imputation techniques to deal with attrition / missing values: Not all participants will use the intervention/comparator as intended and attrition is typically high in ehealth trials. Specify how participants who did not use the application or dropped out from the trial were treated in the statistical analysis (a complete case analysis is strongly discouraged, and simple imputation techniques such as LOCF may also be problematic [4]).

	1	2	3	4	5	
subitem not at all important	○	○	○	◉	○	essential

Xóa lựa chọn

Does your paper address subitem 12a-i? *

Copy and paste relevant sections from the manuscript (include quotes in quotation marks "like this" to indicate direct quotes from your manuscript), or elaborate on this item by providing additional information not in the ms, or briefly explain why the item is not applicable/relevant for your study

"To handle missing data, we used the listwise deletion method to clean the data before analysis."

12b) Methods for additional analyses, such as subgroup analyses and adjusted analyses

Does your paper address CONSORT subitem 12b? *

Copy and paste relevant sections from the manuscript (include quotes in quotation marks "like this" to indicate direct quotes from your manuscript), or elaborate on this item by providing additional information not in the ms, or briefly explain why the item is not applicable/relevant for your study

"Potential covariates for full models of change in risk behaviors among participants included individual characteristics, health status, and adherence. To test our hypotheses, we used multilevel mixed effects linear regression analysis to account for correlations of repeated-measures data for all these scales. Multivariate logistic regression was used to determine factors associated with the change level of three risk behaviors (drinking, smoking, and drug use). A P value <.05 was considered statistically significant."

X26) REB/IRB Approval and Ethical Considerations [recommended as subheading under "Methods"] (not a CONSORT item)

X26-i) Comment on ethics committee approval

	1	2	3	4	5	
subitem not at all important	○	○	○	○	◉	essential

Xóa lựa chọn

Does your paper address subitem X26-i?

Copy and paste relevant sections from the manuscript (include quotes in quotation marks "like this" to indicate direct quotes from your manuscript), or elaborate on this item by providing additional information not in the ms, or briefly explain why the item is not applicable/relevant for your study

"The protocol of this study was approved by the institutional review board of Hanoi Medical University (code: 18NCS17/HDDDDHYHN)."

x26-ii) Outline informed consent procedures

Outline informed consent procedures e.g., if consent was obtained offline or online (how? Checkbox, etc.?), and what information was provided (see 4a-ii). See [6] for some items to be included in informed consent documents.

	1	2	3	4	5	
subitem not at all important	○	○	○	○	◉	essential

Xóa lựa chọn

Does your paper address subitem X26-ii?

Copy and paste relevant sections from the manuscript (include quotes in quotation marks "like this" to indicate direct quotes from your manuscript), or elaborate on this item by providing additional information not in the ms, or briefly explain why the item is not applicable/relevant for your study

"All participants agreed to participate in this study through a written informed consent form."

X26-iii) Safety and security procedures

Safety and security procedures, incl. privacy considerations, and any steps taken to reduce the likelihood or detection of harm (e.g., education and training, availability of a hotline)

	1	2	3	4	5	
subitem not at all important	○	○	○	○	◉	essential

Xóa lựa chọn

Does your paper address subitem X26-iii?

Copy and paste relevant sections from the manuscript (include quotes in quotation marks "like this" to indicate direct quotes from your manuscript), or elaborate on this item by providing additional information not in the ms, or briefly explain why the item is not applicable/relevant for your study

"There was no risk to the patients participating in this study. The information they provided was completely anonymous. The researchers and staff participating in the study signed a commitment not to disclose information collected during the study without the consent of the participants. The collected information is kept confidential and is only used for research purposes, not for other purposes. The data are encrypted to ensure confidentiality of the information."

RESULTS

13a) For each group, the numbers of participants who were randomly assigned, received intended treatment, and were analysed for the primary outcome

NPT: The number of care providers or centers performing the intervention in each group and the number of patients treated by each care provider in each center

Does your paper address CONSORT subitem 13a? *

Copy and paste relevant sections from the manuscript (include quotes in quotation marks "like this" to indicate direct quotes from your manuscript), or elaborate on this item by providing additional information not in the ms, or briefly explain why the item is not applicable/relevant for your study

"By the end of the sample recruitment period, a total of 495 patients were recruited and a baseline assessment was performed [16,17]. Participants were then randomized into the intervention arm (n=248) and the control arm (n=247) by a computer software. After the randomization phase, 65 participants were excluded, including 5 from the intervention arm and 60 from the control arm with the following reasons: family issues (n=2), transferred to other clinics (n=3), used other HIV-assist smartphone apps (n=55), stopped participating in the study (n=2), and technical problems with the smartphone (n=4). A total of 428 HIV patients fulfilled the inclusion criteria and participated in the intervention phases, including 243 patients in the intervention arm and 187 patients in the control arm. Five patients in the intervention group did not complete regular follow-ups after 1 month of the intervention and were excluded. Finally, data of 238 patients in the intervention arm and 187 patients in the control arm were analyzed."

13b) For each group, losses and exclusions after randomisation, together with reasons

Does your paper address CONSORT subitem 13b? (NOTE: Preferably, this is shown in a CONSORT flow diagram) *

Copy and paste relevant sections from the manuscript (include quotes in quotation marks "like this" to indicate direct quotes from your manuscript), or elaborate on this item by providing additional information not in the ms, or briefly explain why the item is not applicable/relevant for your study

Consort flow diagram shown in Figure 1.

13b-i) Attrition diagram

Strongly recommended: An attrition diagram (e.g., proportion of participants still logging in or using the intervention/comparator in each group plotted over time, similar to a survival curve) or other figures or tables demonstrating usage/dose/engagement.

	1	2	3	4	5	
subitem not at all important	○	○	○	○	○	essential

Does your paper address subitem 13b-i?

Copy and paste relevant sections from the manuscript or cite the figure number if applicable (include quotes in quotation marks "like this" to indicate direct quotes from your manuscript), or elaborate on this item by providing additional information not in the ms, or briefly explain why the item is not applicable/relevant for your study

Attrition diagra shown in Figure 1.

14a) Dates defining the periods of recruitment and follow-up

Does your paper address CONSORT subitem 14a? *

Copy and paste relevant sections from the manuscript (include quotes in quotation marks "like this" to indicate direct quotes from your manuscript), or elaborate on this item by providing additional information not in the ms, or briefly explain why the item is not applicable/relevant for your study

"This was a randomized controlled trial including participants recruited from outpatient HIV clinics at Bach Mai Hospital and Ha Dong General Hospital from March 2018 to December 2019."
"These data were collected at baseline and at 1-month and 3-month follow-ups during their ART medication visits with doctors."

14a-i) Indicate if critical “secular events” fell into the study period

Indicate if critical “secular events” fell into the study period, e.g., significant changes in Internet resources available or “changes in computer hardware or Internet delivery resources”

	1	2	3	4	5	
subitem not at all important	(●)	○	○	○	○	essential

Xóa lựa chọn

Does your paper address subitem 14a-i?

Copy and paste relevant sections from the manuscript (include quotes in quotation marks "like this" to indicate direct quotes from your manuscript), or elaborate on this item by providing additional information not in the ms, or briefly explain why the item is not applicable/relevant for your study

None recorded. This study did not have secular events.

14b) Why the trial ended or was stopped (early)

Does your paper address CONSORT subitem 14b? *

Copy and paste relevant sections from the manuscript (include quotes in quotation marks "like this" to indicate direct quotes from your manuscript), or elaborate on this item by providing additional information not in the ms, or briefly explain why the item is not applicable/relevant for your study

Not applicable. The trial was not ended or stopped early.

15) A table showing baseline demographic and clinical characteristics for each group
NPT: When applicable, a description of care providers (case volume, qualification, expertise, etc.) and centers (volume) in each group

Does your paper address CONSORT subitem 15? *

Copy and paste relevant sections from the manuscript (include quotes in quotation marks "like this" to indicate direct quotes from your manuscript), or elaborate on this item by providing additional information not in the ms, or briefly explain why the item is not applicable/relevant for your study

Table 1 and Appendix 1 presented baseline demographic and clinical characteristics.

15-i) Report demographics associated with digital divide issues

In ehealth trials it is particularly important to report demographics associated with digital divide issues, such as age, education, gender, social-economic status, computer/Internet/ehealth literacy of the participants, if known.

	1	2	3	4	5	
subitem not at all important	○	○	◉	○	○	essential

Xóa lựa chọn

Does your paper address subitem 15-i? *

Copy and paste relevant sections from the manuscript (include quotes in quotation marks "like this" to indicate direct quotes from your manuscript), or elaborate on this item by providing additional information not in the ms, or briefly explain why the item is not applicable/relevant for your study

Do not apply. Participants have been trained by doctors and they can contact the doctors if they need assistance.

16) For each group, number of participants (denominator) included in each analysis and whether the analysis was by original assigned groups

16-i) Report multiple “denominators” and provide definitions

Report multiple “denominators” and provide definitions: Report N’s (and effect sizes) “across a range of study participation [and use] thresholds” [1], e.g., N exposed, N consented, N used more than x times, N used more than y weeks, N participants “used” the intervention/comparator at specific pre-defined time points of interest (in absolute and relative numbers per group). Always clearly define “use” of the intervention.

	1	2	3	4	5	
subitem not at all important	○	○	◉	○	○	essential

Xóa lựa chọn

Does your paper address subitem 16-i? *

Copy and paste relevant sections from the manuscript (include quotes in quotation marks "like this" to indicate direct quotes from your manuscript), or elaborate on this item by providing additional information not in the ms, or briefly explain why the item is not applicable/relevant for your study

The information was presented in Table 2, Table 3, Table 4, and Appendix 1. All participants were analyzed in the study arm they were randomized.

16-ii) Primary analysis should be intent-to-treat

Primary analysis should be intent-to-treat, secondary analyses could include comparing only “users”, with the appropriate caveats that this is no longer a randomized sample (see 18-i).

	1	2	3	4	5	
subitem not at all important	○	○	◉	○	○	essential

Xóa lựa chọn

Does your paper address subitem 16-ii?

Copy and paste relevant sections from the manuscript (include quotes in quotation marks "like this" to indicate direct quotes from your manuscript), or elaborate on this item by providing additional information not in the ms, or briefly explain why the item is not applicable/relevant for your study

Primary analysis was intent-to-treat.

17a) For each primary and secondary outcome, results for each group, and the estimated effect size and its precision (such as 95% confidence interval)

Does your paper address CONSORT subitem 17a? *

Copy and paste relevant sections from the manuscript (include quotes in quotation marks "like this" to indicate direct quotes from your manuscript), or elaborate on this item by providing additional information not in the ms, or briefly explain why the item is not applicable/relevant for your study

This study measure the difference from baseline value. This detail was prensented in Table 3.

17a-i) Presentation of process outcomes such as metrics of use and intensity of use

In addition to primary/secondary (clinical) outcomes, the presentation of process outcomes such as metrics of use and intensity of use (dose, exposure) and their operational definitions is critical. This does not only refer to metrics of attrition (13-b) (often a binary variable), but also to more continuous exposure metrics such as “average session length”. These must be accompanied by a technical description how a metric like a “session” is defined (e.g., timeout after idle time) [1] (report under item 6a).

	1	2	3	4	5	
subitem not at all important	○	○	○	○	○	essential

Does your paper address subitem 17a-i?

Copy and paste relevant sections from the manuscript (include quotes in quotation marks "like this" to indicate direct quotes from your manuscript), or elaborate on this item by providing additional information not in the ms, or briefly explain why the item is not applicable/relevant for your study

Not applicable. Participants in the intervention group were reminded daily. All participants in the intervention group participated until the end.

17b) For binary outcomes, presentation of both absolute and relative effect sizes is recommended

Does your paper address CONSORT subitem 17b? *

Copy and paste relevant sections from the manuscript (include quotes in quotation marks "like this" to indicate direct quotes from your manuscript), or elaborate on this item by providing additional information not in the ms, or briefly explain why the item is not applicable/relevant for your study

Outcomes of this study are continuous variables. The difference from the baseline value was calculated.

18) Results of any other analyses performed, including subgroup analyses and adjusted analyses, distinguishing pre-specified from exploratory

Does your paper address CONSORT subitem 18? *

Copy and paste relevant sections from the manuscript (include quotes in quotation marks "like this" to indicate direct quotes from your manuscript), or elaborate on this item by providing additional information not in the ms, or briefly explain why the item is not applicable/relevant for your study

Multilevel mixed effects linear regression to identify factors related to change in adherence and symptom management self-efficacy among participants and Logistic regression to identify factors related to change in risk behaviors among participants were performed in Table 5 and Table 6.

18-i) Subgroup analysis of comparing only users

A subgroup analysis of comparing only users is not uncommon in ehealth trials, but if done, it must be stressed that this is a self-selected sample and no longer an unbiased sample from a randomized trial (see 16-iii).

	1	2	3	4	5	
subitem not at all important	○	○	○	◉	○	essential

Xóa lựa chọn

Does your paper address subitem 18-i?

Copy and paste relevant sections from the manuscript (include quotes in quotation marks "like this" to indicate direct quotes from your manuscript), or elaborate on this item by providing additional information not in the ms, or briefly explain why the item is not applicable/relevant for your study

We examined the change of adherence, HIV treatment adherence self-efficacy, and symptom management self-efficacy among HIV patients at 1 and 3 months after starting the intervention (Table 3) as well as the level of change of risk behaviors at the 3-month follow-up (Table 4).

19) All important harms or unintended effects in each group
(for specific guidance see CONSORT for harms)

Does your paper address CONSORT subitem 19? *

Copy and paste relevant sections from the manuscript (include quotes in quotation marks "like this" to indicate direct quotes from your manuscript), or elaborate on this item by providing additional information not in the ms, or briefly explain why the item is not applicable/relevant for your study

"There was no risk to the patients participating in this study."

19-i) Include privacy breaches, technical problems

Include privacy breaches, technical problems. This does not only include physical “harm” to participants, but also incidents such as perceived or real privacy breaches [1], technical problems, and other unexpected/unintended incidents. “Unintended effects” also includes unintended positive effects [2].

	1	2	3	4	5	
subitem not at all important	○	○	○	○	◉	essential

Xóa lựa chọn

Does your paper address subitem 19-i?

Copy and paste relevant sections from the manuscript (include quotes in quotation marks "like this" to indicate direct quotes from your manuscript), or elaborate on this item by providing additional information not in the ms, or briefly explain why the item is not applicable/relevant for your study

"The information they provided was completely anonymous. The researchers and staff participating in the study signed a commitment not to disclose information collected during the study without the consent of the participants. The collected information is kept confidential and is only used for research purposes, not for other purposes. The data are encrypted to ensure confidentiality of the information."

19-ii) Include qualitative feedback from participants or observations from staff/researchers

Include qualitative feedback from participants or observations from staff/researchers, if available, on strengths and shortcomings of the application, especially if they point to unintended/unexpected effects or uses. This includes (if available) reasons for why people did or did not use the application as intended by the developers.

	1	2	3	4	5	
subitem not at all important	○	○	○	○	○	essential

Does your paper address subitem 19-ii?

Copy and paste relevant sections from the manuscript (include quotes in quotation marks "like this" to indicate direct quotes from your manuscript), or elaborate on this item by providing additional information not in the ms, or briefly explain why the item is not applicable/relevant for your study

This study did not include qualitative feedack from participants.

DISCUSSION

22) Interpretation consistent with results, balancing benefits and harms, and considering other relevant evidence

NPT: In addition, take into account the choice of the comparator, lack of or partial blinding, and unequal expertise of care providers or centers in each group

22-i) Restate study questions and summarize the answers suggested by the data, starting with primary outcomes and process outcomes (use)

Restate study questions and summarize the answers suggested by the data, starting with primary outcomes and process outcomes (use).

	1	2	3	4	5	
subitem not at all important	○	○	○	○	◉	essential

Xóa lựa chọn

Does your paper address subitem 22-i? *

Copy and paste relevant sections from the manuscript (include quotes in quotation marks "like this" to indicate direct quotes from your manuscript), or elaborate on this item by providing additional information not in the ms, or briefly explain why the item is not applicable/relevant for your study

"Our study revealed that those who used the app had increased adherence and a higher level of self-efficacy in terms of symptom management. However, the effectiveness of this app in changing risk behaviors such as smoking, alcohol use, and drug use was limited."

22-ii) Highlight unanswered new questions, suggest future research

Highlight unanswered new questions, suggest future research.

	1	2	3	4	5	
subitem not at all important	○	○	○	◉	○	essential

Xóa lựa chọn

Does your paper address subitem 22-ii?

Copy and paste relevant sections from the manuscript (include quotes in quotation marks "like this" to indicate direct quotes from your manuscript), or elaborate on this item by providing additional information not in the ms, or briefly explain why the item is not applicable/relevant for your study

"Certain limitations also exist in our study. With respect to the confidentiality of HIV diagnosis and medical records, we were not able to preidentify the survey sample. Instead, the sample of participants was identified using convenience sampling and was subsequently randomized. The randomization method could not remove all underlying differences among participants assigned to the experimental or control conditions. There were also differences in educational levels, marital status, age, and duration of ART between the two groups. In addition, we acknowledge that ART outcomes will reduce transmission as well. However, we did not include sexual behaviors in our assessments, as we realized during the pilot trial that this remains a sensitive topic among Vietnamese people living with HIV, which could have affected the compliance to the app-based intervention since they were afraid of being traced and a loss of confidentiality. Furthermore, the follow-up period in our study was 3 months, while the ideal follow-up period should be 6 months or longer to fully determine the sustainability of treatment improvements."

20) Trial limitations, addressing sources of potential bias, imprecision, and, if relevant, multiplicity of analyses

20-i) Typical limitations in ehealth trials

Typical limitations in ehealth trials: Participants in ehealth trials are rarely blinded. Ehealth trials often look at a multiplicity of outcomes, increasing risk for a Type I error. Discuss biases due to non-use of the intervention/usability issues, biases through informed consent procedures, unexpected events.

	1	2	3	4	5	
subitem not at all important	○	○	○	◉	○	essential

Xóa lựa chọn

Does your paper address subitem 20-i? *

Copy and paste relevant sections from the manuscript (include quotes in quotation marks "like this" to indicate direct quotes from your manuscript), or elaborate on this item by providing additional information not in the ms, or briefly explain why the item is not applicable/relevant for your study

"Certain limitations also exist in our study. With respect to the confidentiality of HIV diagnosis and medical records, we were not able to preidentify the survey sample. Instead, the sample of participants was identified using convenience sampling and was subsequently randomized. The randomization method could not remove all underlying differences among participants assigned to the experimental or control conditions. There were also differences in educational levels, marital status, age, and duration of ART between the two groups. In addition, we acknowledge that ART outcomes will reduce transmission as well. However, we did not include sexual behaviors in our assessments, as we realized during the pilot trial that this remains a sensitive topic among Vietnamese people living with HIV, which could have affected the compliance to the app-based intervention since they were afraid of being traced and a loss of confidentiality. Furthermore, the follow-up period in our study was 3 months, while the ideal follow-up period should be 6 months or longer to fully determine the sustainability of treatment improvements."

21) Generalisability (external validity, applicability) of the trial findings

NPT: External validity of the trial findings according to the intervention, comparators, patients, and care providers or centers involved in the trial

21-i) Generalizability to other populations

Generalizability to other populations: In particular, discuss generalizability to a general Internet population, outside of a RCT setting, and general patient population, including applicability of the study results for other organizations

	1	2	3	4	5	
subitem not at all important	○	○	●	○	○	essential

Xóa lựa chọn

Does your paper address subitem 21-i?

Copy and paste relevant sections from the manuscript (include quotes in quotation marks "like this" to indicate direct quotes from your manuscript), or elaborate on this item by providing additional information not in the ms, or briefly explain why the item is not applicable/relevant for your study

Convenience sampling technique was limited the generalizability to other populations.

21-ii) Discuss if there were elements in the RCT that would be different in a routine application setting

Discuss if there were elements in the RCT that would be different in a routine application setting (e.g., prompts/reminders, more human involvement, training sessions or other co-interventions) and what impact the omission of these elements could have on use, adoption, or outcomes if the intervention is applied outside of a RCT setting.

	1	2	3	4	5	
subitem not at all important	○	○	◉	○	○	essential

Xóa lựa chọn

Does your paper address subitem 21-ii?

Copy and paste relevant sections from the manuscript (include quotes in quotation marks "like this" to indicate direct quotes from your manuscript), or elaborate on this item by providing additional information not in the ms, or briefly explain why the item is not applicable/relevant for your study

"The intervention group was given access to the eCARE app through medical staff at the treatment facility." However, in a routine setting might includ those participants excluded in this study such as those who use any other HIV-assist smartphone app at the time of the study, or had cognitive impairments or disabilities that would hinder the use of our app.

OTHER INFORMATION

23) Registration number and name of trial registry

Does your paper address CONSORT subitem 23? *

Copy and paste relevant sections from the manuscript (include quotes in quotation marks "like this" to indicate direct quotes from your manuscript), or elaborate on this item by providing additional information not in the ms, or briefly explain why the item is not applicable/relevant for your study

"Thai Clinical Trials Registry TCTR20220928003;
https://www.thaiclinicaltrials.org/show/TCTR20220928003."

24) Where the full trial protocol can be accessed, if available

Does your paper address CONSORT subitem 24? *

Cite a Multimedia Appendix, other reference, or copy and paste relevant sections from the manuscript (include quotes in quotation marks "like this" to indicate direct quotes from your manuscript), or elaborate on this item by providing additional information not in the ms, or briefly explain why the item is not applicable/relevant for your study

Trial protocol is not freely avaiable.

25) Sources of funding and other support (such as supply of drugs), role of funders

Does your paper address CONSORT subitem 25? *

Copy and paste relevant sections from the manuscript (include quotes in quotation marks "like this" to indicate direct quotes from your manuscript), or elaborate on this item by providing additional information not in the ms, or briefly explain why the item is not applicable/relevant for your study

"This study received funding from Bach Mai Medical College, Bach Mai Hospital. The article process charge of this paper is supported by NUS Department of Psychological Medicine (R-177-000-100-001/R-177-000-003-001) and NUS iHeathtech Other Operating Expenses (R-722-000-004-731)."

X27) Conflicts of Interest (not a CONSORT item)

X27-i) State the relation of the study team towards the system being evaluated

In addition to the usual declaration of interests (financial or otherwise), also state the relation of the study team towards the system being evaluated, i.e., state if the authors/evaluators are distinct from or identical with the developers/sponsors of the intervention.

	1	2	3	4	5	
subitem not at all important	○	○	○	◉	○	essential

Xóa lựa chọn

Does your paper address subitem X27-i?

Copy and paste relevant sections from the manuscript (include quotes in quotation marks "like this" to indicate direct quotes from your manuscript), or elaborate on this item by providing additional information not in the ms, or briefly explain why the item is not applicable/relevant for your study

This study did not have conflicts of Interest.

About the CONSORT EHEALTH checklist

As a result of using this checklist, did you make changes in your manuscript? *

- ( ) yes, major changes
- (•) yes, minor changes
- ( ) no

What were the most important changes you made as a result of using this checklist?

Changes in the methods and results sections.

How much time did you spend on going through the checklist INCLUDING making changes in your manuscript *

I spend about 4 hours to make changes.

As a result of using this checklist, do you think your manuscript has improved? *

- (•) yes
- ( ) no
- ( ) Mục khác:

Would you like to become involved in the CONSORT EHEALTH group?
This would involve for example becoming involved in participating in a workshop and writing an "Explanation and Elaboration" document

- ○ yes
- ◉ no
- ○ Mục khác:

Xóa lựa chọn

Any other comments or questions on CONSORT EHEALTH

I do not have any commnents.

STOP - Save this form as PDF before you click submit
To generate a record that you filled in this form, we recommend to generate a PDF of this page (on a Mac, simply select "print" and then select "print as PDF") before you submit it.

When you submit your (revised) paper to JMIR, please upload the PDF as supplementary file.

Don't worry if some text in the textboxes is cut off, as we still have the complete information in our database. Thank you!

Final step: Click submit !
Click submit so we have your answers in our database!

Gửi Xóa hết câu trả lời

Không bao giờ gửi mật khẩu thông qua Google Biểu mẫu.

Nội dung này không phải do Google tạo ra hay xác nhận. Báo cáo Lạm dụng - Điều khoản Dịch vụ - Chính sách

quyền riêng tư

Google Biểu mẫu
